# Dermal tattoos in biomedicine: Emerging platforms for sensing and therapy

**DOI:** 10.1016/j.mtbio.2026.103511

**Published:** 2026-07-30

**Authors:** Reyhaneh Shakibi, Ali Golkar, Hamid Reza Bagheri, Mohammad Ali Khayamian

**Affiliations:** a Institute of Biochemistry and Biophysics (IBB), University of Tehran, Tehran, Iran; b Integrated Biophysics and Bioengineering Lab (iBL), Institute of Biochemistry and Biophysics, University of Tehran, Tehran, Iran

**Keywords:** Dermal tattoo, Biomedical sensing, Colorimetric biosensors, Wearable diagnostics, Bioelectronic interfaces

## Abstract

Dermal tattoos are emerging as a novel class of skin-integrated biomedical platforms that extend beyond body decoration into functional sensing, monitoring, and therapeutic applications. In contrast to conventional biomedical devices, wearable systems, and epidermal electronics, dermal tattoos reside within the dermis, where they can access interstitial fluid, achieve stable tissue integration, and remain protected from surface contamination and mechanical disruption. Although this field is still in its infancy, recent studies have demonstrated the potential of dermal tattoos for a broad range of biomedical uses, particularly in sensing and diagnostics. Reported platforms include colorimetric, fluorescent, photoresponsive, living, and electrophysiological dermal tattoo systems for monitoring biomarkers, electrolytes, ultraviolet exposure, and physiological electrical signals. More recently, dermal tattoos have also begun to expand into treatment, including self-powered bioelectronic systems for wound healing and transient bioresorbable therapeutic interfaces. This review summarizes recent advances in dermal tattoos for biomedicine, focusing on sensing and therapy, along with their materials, fabrication methods, and readout strategies. It also compares dermal tattoos with conventional, wearable, and epidermal systems, and discusses their key opportunities, challenges, and future directions. However, most reported dermal tattoo platforms remain at the proof-of-concept stage, and their clinical translation is still limited by scarce in vivo validation, lack of standardized benchmarking, and limited long-term safety data.

## Graphical abstract

**Figure:**
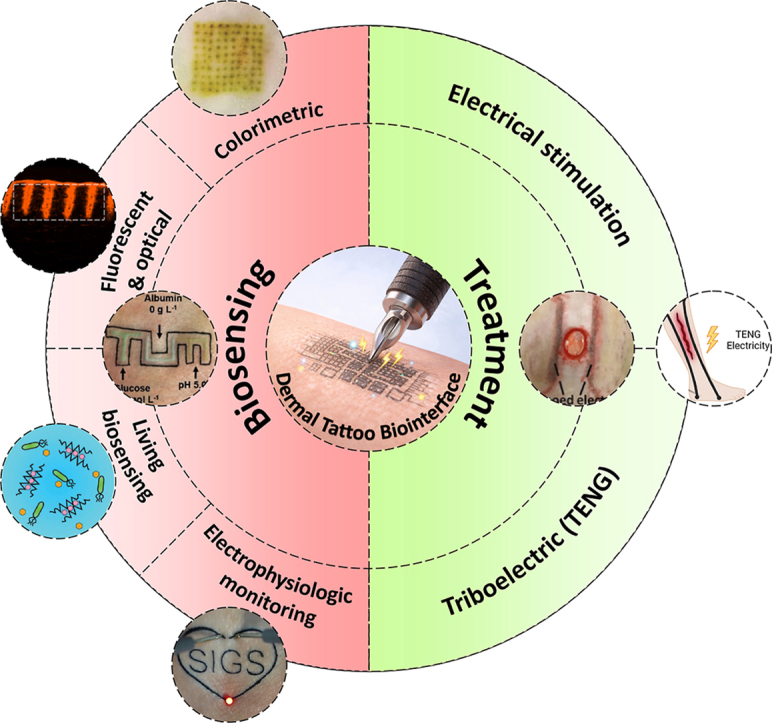

## 1 Introduction

**Fig. 1:**
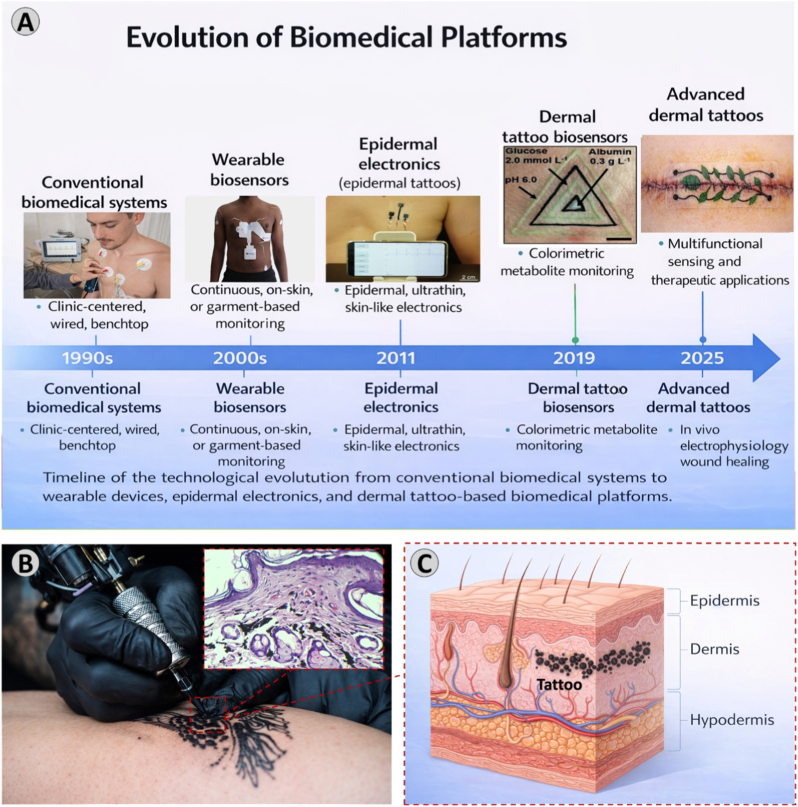
Overview of dermal tattoos as an emerging biomedical platform. (A) Timeline showing the evolution from conventional biomedical systems to wearable devices, epidermal electronics, and dermal tattoo-based platforms [[3], [4], [5], [6]]. (B) Representative tattooing process using a conventional tattoo gun, with inset showing histological evidence of pigment deposition in skin. (C) Schematic cross-section of skin showing tattoo localization within the dermis beneath the epidermis.

The growing demand for personalized healthcare has accelerated the development of biomedical platforms capable of providing continuous sensing, real-time monitoring, and localized treatment [1]. In recent years, there has been a clear shift from intermittent, clinic-centered assessment toward body-integrated systems that can operate directly at the point of care [2] (Fig. 1-A). Such platforms are particularly attractive for applications including metabolic monitoring [7,8], physiological recording [9,10], wound management [11,12], and therapeutic intervention [13,14], where minimally invasive and patient-friendly designs are highly desirable [15].

Traditional biomedical sensing and treatment platforms have played a foundational role in modern medicine due to their analytical accuracy, controllability, and established clinical performance. Examples include benchtop biochemical analyzers for metabolite quantification, wired electrophysiological systems for ECG or EMG recording [16], and externally powered electrical stimulation devices used for wound or cancer care [17]. Despite their effectiveness, these conventional systems are often bulky, rigid, and dependent on external instrumentation or wired connections, which limits portability, reduces patient comfort, and restricts their use in continuous daily-life settings [18,19].

To address these limitations, wearable biomedical devices emerged as the next major step in the evolution of healthcare technologies [20,21]. Flexible patches [22,23], wrist-worn biosensors [24,25], textile-integrated devices [26,27], and skin-mounted systems [28,29] enabled improved portability and more continuous interaction with the human body. These platforms significantly expanded the possibility of ambulatory monitoring and mobile therapy. However, many wearable systems still remain relatively bulky or mechanically mismatched with soft tissue, and long-term use can be accompanied by issues such as discomfort, device displacement, battery replacement, limited conformability, and reduced signal reliability [30,31]. However, several recent reviews have discussed strategies for overcoming the limitations of these next-generation medical devices [[32], [33], [34], [35]].

The development of epidermal electronics [36,37] and electronic tattoos further advanced this concept by bringing biointerfaces into intimate contact with the skin surface [38]. Owing to their ultrathin geometry, softness, and mechanical compliance, epidermal devices can conform closely to the skin and have been widely investigated for electrophysiological monitoring, sweat analysis, strain sensing, and motion tracking [39,40]. Nevertheless, because these systems remain surface-bound, they still suffer from important drawbacks. Sweat, friction, repeated deformation, and routine physical activity can weaken adhesion, induce motion artifacts, and shorten device lifetime [[41], [42], [43]]. As a result, although epidermal platforms represent a major improvement over conventional wearables, they do not fully solve the challenges of long-term stability, robust biointegration, and uninterrupted functionality (Table 1).

**Table 1:** Qualitative comparison of conventional biomedical platforms, wearable devices, epidermal systems and dermal tattoos.

Parameter	Conventional platforms	Wearable devices	Epidermal tattoos/epidermal electronics	Dermal tattoos
Typical location	External, off-body, or loosely body-coupled	On-body	On the skin surface	Within the dermis
Body integration	Low; usually external or intermittently connected to the body	Moderate; attached to the body surface	High; conformal contact with the skin surface	Potentially high because the functional material is localized inside the dermis
Mechanical conformability	Low to moderate; depends on device geometry and fixation	Moderate; limited by device thickness, substrate rigidity, and attachment method	High; ultrathin and skin-like formats have been reported	High potential, but depends on tattoo material, deposition method, and local tissue response
Access to biological fluid/physiological environment	Usually indirect or sample-dependent, e.g., blood, saliva, sweat, or extracted samples	Often indirect; commonly based on sweat, motion, optical signals, or surface-associated fluids	Surface-associated; mainly interacts with sweat, skin surface, or superficial biointerfaces	Direct access to local dermal ISF, although analyte-specific correlation with blood requires validation
Signal quality during motion	High in controlled clinical settings, but can be affected by motion during daily-life use	Variable; affected by device displacement, fixation, and skin deformation	Often improved compared with rigid wearables because of conformal skin contact, but still surface-bound	Promising for selected applications because intradermal localization may reduce surface displacement effects; standardized motion benchmarking remains limited
Susceptibility to motion artifacts	Can be high during ambulatory or daily-life use	Moderate to high, depending on device fixation and activity level	Generally lower than bulky wearables, but still affected by skin strain, adhesion loss, and surface deformation	Expected to be reduced in some systems because the functional material is beneath the skin surface, but direct quantitative comparisons remain scarce
Exposure to sweat, friction, and environment	Variable; depends on device and sampling route	High; exposed to sweat, humidity, clothing friction, and environmental disturbance	High; located directly on the skin surface and exposed to abrasion, washing, and exfoliation	Lower for intradermal components because they are protected by the epidermis, although external readout components may remain exposed
Need for external hardware	Usually high; often requires benchtop, handheld, or clinical instrumentation	Moderate to high; commonly requires batteries, electronics, wireless modules, or smartphones	Moderate; often requires external readout electronics, optical systems, or wireless modules	Low to moderate depending on readout strategy; optical systems may require a camera or reader, while active electronic systems still require external components
User comfort	Often limited during continuous use because of cables, adhesives, sampling, or bulky instrumentation	Improved compared with conventional systems, but depends on device size, battery, adhesion, and wear duration	Generally high because of ultrathin, lightweight, and conformal construction	Potentially high after implantation, but depends on pain, insertion method, material biocompatibility, permanence, and user acceptance
Aesthetic compatibility	Low; usually visible and medical-looking	Low to moderate; depends on size, placement, and design	Moderate; can be thin or visually subtle, but remains surface-visible	High potential because tattoos can be personalized, decorative, invisible, fluorescent, or transient
Potential for continuous monitoring	Limited outside clinical settings	Moderate to high; already demonstrated in several commercial wearable platforms	High for many skin-mounted electronic systems, although adhesion and skin renewal limit long-term operation	Promising, especially for long-term optical or biochemical monitoring, but continuous clinical validation remains limited
Potential for therapeutic integration	High in established clinical systems such as stimulators, infusion pumps, or closed-loop devices	Moderate; demonstrated in selected wearable drug-delivery or stimulation systems	Emerging; integration with stimulation or drug delivery remains technically challenging	Very early but promising; currently supported by limited proof-of-concept evidence
Invasiveness	Noninvasive to invasive, depending on sampling or device type	Mostly noninvasive	Noninvasive; attached to the skin surface	Minimally invasive because functional materials are deposited into the dermis
Retention/operational lifetime	Device-dependent; can be long in controlled clinical settings	Limited by battery life, adhesion, maintenance, and user compliance	Limited by adhesion, sweat, washing, and skin exfoliation	Can be transient or long-term depending on material design, biodegradation, immune response, and pigment retention
Risk of external contamination	Moderate to high depending on sampling route and handling	High for sweat- and skin-contact sensors	High because sensing interfaces remain on the skin surface	Potentially lower for intradermal components, but implantation introduces sterility and biocompatibility requirements
Suitability for personalization	Low to moderate; usually standardized device formats	Moderate; placement and software calibration may be adjustable	Moderate; geometry and sensor layout can be customized	High potential through personalized tattoo geometry, color, visibility, chemistry, and lifetime
Potential for bioresorbability	No	Limited	Limited but possible using biodegradable substrates	Promising for transient dermal materials, biodegradable pigments, and resorbable microneedle-based systems
Current translational maturity	High for many established clinical platforms	Moderate to high for several commercial systems	Moderate; many systems remain research-stage but the field is mature relative to dermal tattoos	Early-stage; most studies remain proof-of-concept, with limited clinical validation

In this context, dermal tattoos are emerging as a new generation of skin-integrated biomedical interfaces [[3], [4], [5],[44], [45], [46], [47], [48]]. In contrast to epidermal devices that rest on the outer skin surface, dermal tattoos are embedded beneath the epidermis [49] and therefore offer a more stable and mechanically protected interface with the body. This deeper placement may improve electrophysiological signal quality by bypassing the highly resistive stratum corneum and reducing motion-related signal disturbance, although direct dermal-versus-epidermal impedance benchmarking remains limited. In addition, dermal tattoos offer unique practical and aesthetic advantages [50]: they can be highly conformal, minimally obtrusive, visually personalized, and, depending on the materials used, either long-lasting or transient. These features make them especially attractive for biomedical applications in which both functionality and user acceptance are important.

Although the field is still in its infancy, the currently reported biomedical applications of dermal tattoos already reveal remarkable diversity. Most published studies have focused on sensing and diagnostic functions, including colorimetric metabolite detection [3,44], fluorescent and optical biosensing [[46], [47], [48]], living biosensors [45] based on engineered bacteria, and electrophysiological monitoring [4]. In contrast, therapeutic applications remain very limited, with only rare examples extending dermal tattoos into treatment-oriented platforms such as bioelectronic stimulation or self-powered therapeutic systems [5]. This imbalance suggests that dermal tattoos have so far been explored primarily as diagnostic interfaces, while their potential in treatment and closed-loop biomedical systems remains largely underdeveloped.

At the same time, dermal tattoo studies remain exceptionally scarce compared with the much broader fields of conventional wearables and epidermal electronics. The literature is still fragmented across sensing, monitoring, and treatment, and a focused overview of dermal tattoos as a distinct biomedical platform is still lacking. A dedicated review is therefore timely and necessary to organize current advances, compare sensing and therapeutic strategies, examine fabrication methods and material choices, and identify the key opportunities and challenges that will shape the future of this emerging field. In this review, we discuss recent progress in dermal tattoos for biomedicine, with particular emphasis on their roles in sensing, diagnostics, and therapy, and we highlight the future potential of dermal tattoos as next-generation biomedical interfaces. It should be emphasized that dermal tattoo biomedicine is still an emerging and early-stage field; most available studies are proof-of-concept demonstrations performed in ex vivo skin, skin-mimicking models, or small-animal experiments, with very limited clinical validation.

## 2 Anatomical and biological basis of dermal tattoos

In practice, dermal tattoos are most commonly created using a tattoo machine (tattoo gun) (Fig. 1-B), in which a rapidly oscillating needle repeatedly penetrates the skin and delivers ink or functional material beneath the epidermis into the dermis [51]. Depending on the application, the deposited material may be a conventional pigment, a sensing reagent, a fluorescent probe, a living microgel, or a conductive ink. The depth, angle, and density of needle penetration influence the final distribution of the material in the dermis and therefore affect both tattoo appearance and biomedical performance. In some studies, alternative methods such as microneedle patches [44] is used to improve control and reproducibility, but tattoo-gun deposition [5] remains the most familiar and widely recognized route for dermal tattoo formation.

The biomedical potential of dermal tattoos arises from their unique position at the intersection of skin biology, interstitial-fluid access, and long-term material retention. Unlike epidermal devices that remain on the skin surface, dermal tattoos are introduced beneath the epidermis and reside within the dermal layer (Fig. 1-B), where they can interact more directly with the local physiological environment. This under-skin localization gives dermal tattoos a distinctive status among biomedical interfaces: they are more deeply integrated than conventional wearables, yet less invasive than many implantable systems.

Human skin is a multilayered organ composed primarily of the epidermis, dermis, and hypodermis (subcutaneous tissue) (Fig. 1-C) [52] and their role is undisputable in skin-mounted bioelectronics [53]. The epidermis is the outermost layer and functions mainly as a protective barrier against mechanical damage, water loss, and environmental exposure. Its outermost component, the stratum corneum, is especially important in biomedical device design because it creates a major barrier to mass transfer and electrical interfacing [54]. Beneath the epidermis lies the dermis, a connective tissue-rich layer containing fibroblasts, immune cells, extracellular matrix, blood vessels, lymphatics, and nerve endings. Below the dermis is the hypodermis, which consists largely of adipose and loose connective tissue and serves structural, cushioning, and metabolic functions.

Dermal tattoos are typically positioned within the dermis, rather than on the skin surface or in deeper subcutaneous tissue (Fig. 1-C). This location is fundamental to their biomedical value. Because the dermis contains abundant interstitial fluid (ISF) and is closely associated with capillary exchange, it provides access to a physiologically relevant local microenvironment [55]. ISF contains a wide range of clinically relevant biomarkers that can be accessed for sensing applications [56]. Small molecules such as glucose, lactate, urea, and electrolytes (e.g., Na^+^ and K^+^) can readily diffuse from the capillary circulation into the interstitial space and often show concentrations that correlate with those in blood [57]. Larger biomolecules, including albumin, insulin, and cytokines, are also present in ISF but their transport is partially restricted by size and charge-dependent barriers across the capillary endothelium [58]. In addition to metabolic markers, inflammatory mediators such as IL-6, TGF-β, and VEGF may also be detectable in ISF under pathological conditions [59]. ISF contains many analytes of biomedical interest [60] and often reflects systemic biochemical changes, making the dermis an attractive site for minimally invasive sensing. Interstitial fluid contains a wide range of physiologically relevant biomarkers that can potentially be monitored using dermal tattoo–based sensing systems. Typical examples include metabolites such as glucose, whose dermal ISF levels generally correlate well with capillary or venous blood glucose and are commonly examined within clinically relevant ranges of about 50–350 mg/dL (∼2.8–19.4 mM) [61,62]. However, measured values can vary depending on factors such as the extraction technique, sampling site, calibration method, and potential evaporation during collection. Other important ISF metabolites include lactate (∼0.5–5 mM) [63], and urea (∼3–7.5 mM) [64], which provide insights into metabolic activity and tissue perfusion. Electrolytes including sodium (∼135–145 mM) [56,65], potassium (∼3.5–5 mM) and chloride (∼95–110 mM) [66], calcium (∼1–2 mM) [67] are also present in ISF and are important indicators of electrolyte balance and physiological homeostasis. These findings indicate that ISF contains a diverse range of candidate biomarkers that may be useful for disease detection and monitoring. Together, these analytes demonstrate the extensive biochemical information available in dermal interstitial fluid and support its potential as a diagnostic medium for dermal tattoo biosensors. At the same time, the overlying epidermis serves as a natural barrier, protecting the tattooed material from external contamination, sweat, friction, and other environmental influences. The dermis may also provide a favorable setting for material residence. Its extracellular matrix, primarily composed of collagen and elastin fibers, contributes to both mechanical strength and flexibility, while resident cells support tissue maintenance and wound healing. For biomedical tattoos, this environment can help support the implanted material within a soft, compliant, and biologically active tissue. Compared with surface-mounted devices, dermal tattoos may therefore offer improved positional stability, lower risk of detachment, and closer interaction with living tissue.

These anatomical characteristics are highly relevant to both sensing and treatment. For sensing applications, dermal localization enables contact with ISF while protecting the sensing material beneath the skin surface. For electrical and bioelectronic applications, placement within or beneath the epidermal barrier may improve signal quality and improve communication with tissue. Thus, the dermis is not simply the site where tattoo materials happen to reside; it is the key anatomical compartment that makes dermal tattoos especially attractive as biomedical interfaces.

After deposition into the dermis, tattooed inks typically undergo a common biological fate governed by the local wound-healing and foreign-body response [68]. Immediately after tattooing, needle penetration causes mild tissue injury, which triggers acute inflammation and recruitment of immune cells. A portion of the injected material is taken up by macrophages [69] and other phagocytic cells, while another portion remains trapped within the extracellular matrix and among dermal fibroblasts [70]. This combination of cellular uptake and matrix entrapment is largely responsible for the long-term retention of tattoo materials in the skin. Over time, the fate of the ink depends on its composition, particle size, and biodegradability: conventional pigments and many stable functional materials may persist for extended periods, whereas specially designed transient or bioresorbable inks may gradually fade, degrade, or be cleared from the tissue. Thus, the biological fate of dermal tattoos is a balance between retention, redistribution, cellular sequestration, and, in some systems, programmed disappearance. However, the same anatomical advantages introduce variability in insertion depth, local tissue response, and signal path length, all of which can alter sensor performance across subjects and anatomical sites.

## 3 Dermal tattoos for sensing and diagnostics

**Fig. 2:**
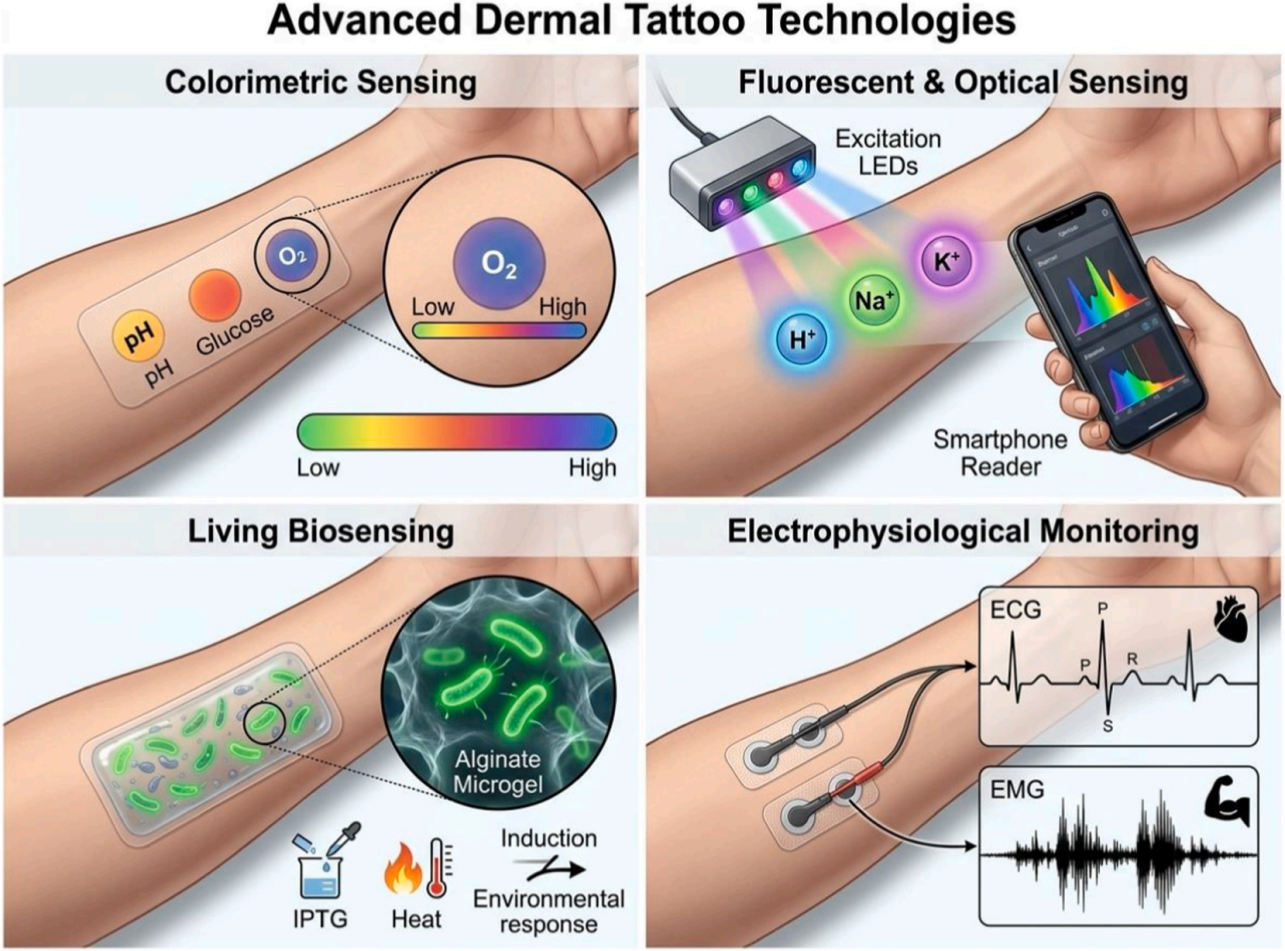
Dermal tattoo-based sensing platforms categorized into colorimetric sensing, fluorescent and optical sensing, living biosensing, and electrophysiological monitoring. These approaches enable biochemical, optical, biological, and electrical interrogation through the dermal interface.

Among the currently reported biomedical applications of dermal tattoos, sensing and diagnostics clearly represent the dominant direction. Most published studies have used dermal tattoos as minimally invasive platforms for detecting biochemical, optical, or electrophysiological signals, while therapeutic applications remain comparatively rare. This strong emphasis on sensing is not surprising, as the dermis offers several intrinsic advantages as a diagnostic interface such as close proximity to local interstitial fluid (ISF), the coverage and protection of the functional tattooed material by epidermis and longer retention and improved mechanical stability. Based on the sensing mechanisms reported to date, dermal tattoo diagnostic platforms can be broadly categorized into colorimetric sensing, fluorescent and optical sensing, living biosensing, and electrophysiological monitoring (Fig. 2).

### 3.1 Colorimetric sensing

Colorimetric sensing represents one of the earliest and most foundational directions in dermal tattoo research. In these systems, the dermal tattoo contains colorimetric reagents that interact with target analytes in the surrounding interstitial fluid, producing a visible color change that reflects the local biochemical state. This approach is particularly attractive because it is conceptually simple, low-cost, potentially equipment-free, and highly compatible with point-of-care use. In addition, the tattoo is protected by the outer skin layers, reducing exposure to sweat and environmental contaminants while maintaining close access to interstitial fluid.

**Fig. 3:**
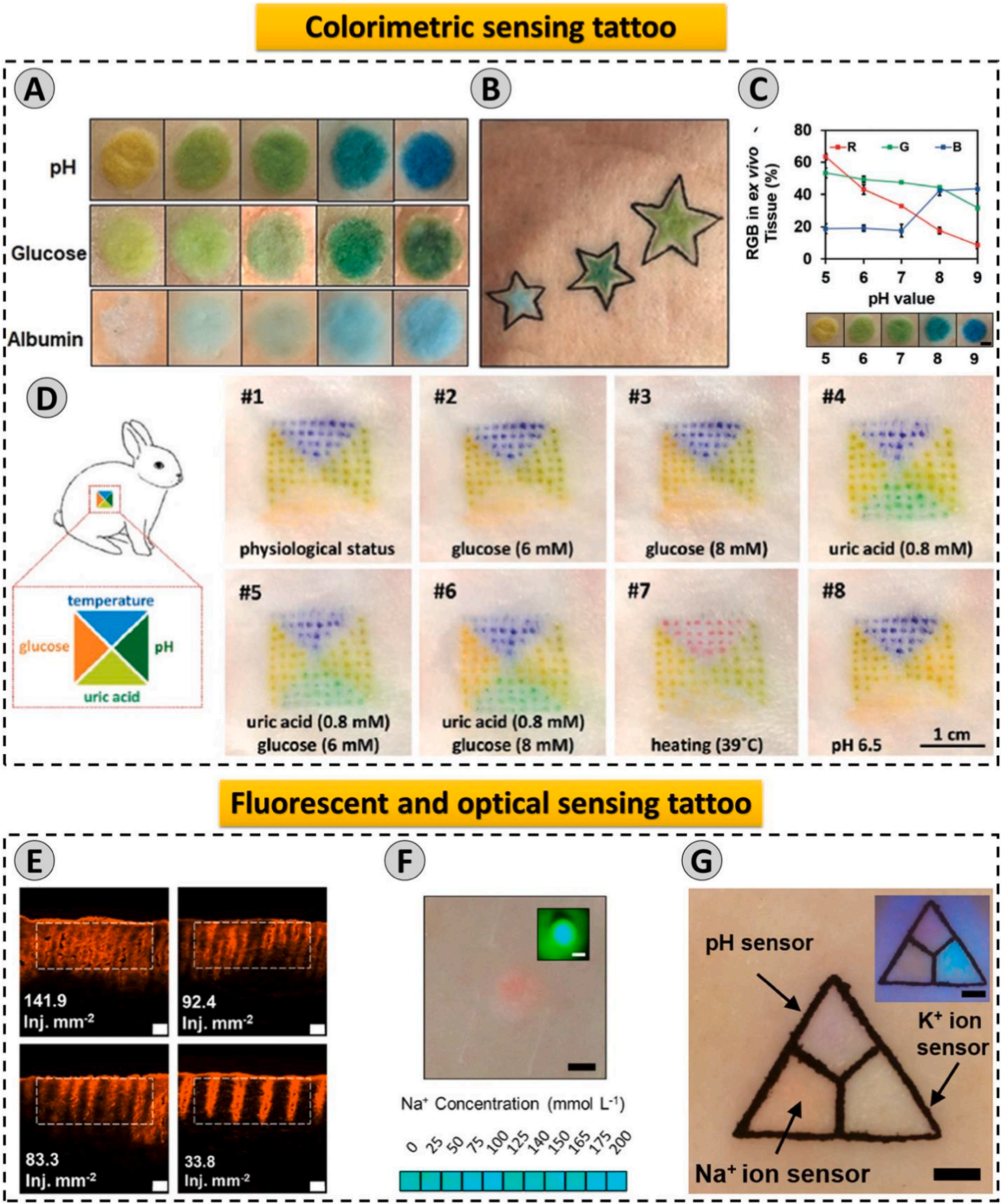
Representative examples of colorimetric and fluorescent/optical dermal tattoo sensing. (A) Colorimetric dermal tattoo responses for pH, glucose and albumin, showing analyte-dependent changes in visible color. (B) Example of an aesthetic colorimetric dermal tattoo pattern integrated into a star-shaped design. (C) Quantitative RGB analysis of the pH-responsive dermal tattoo in ex vivo tissue, with corresponding color changes across different pH values. Adapted with permission from Ref. [3], Yetisen, A. K.; et al. Angew. Chem. 2019, 131, 10616–10623. Copyright © 2019 Wiley-VCH Verlag GmbH & Co. KGaA. (D) Multiplexed colorimetric dermal tattoo layout for simultaneous monitoring of temperature, glucose, uric acid, and pH, together with representative outputs under different physiological or simulated conditions. Adapted from Ref. [44], He et al., Advanced Science, 2021. https://doi.org/10.1002/advs.202103030. Licensed under CC BY 4.0. (E) Fluorescent cross-sectional images showing controlled dermal tattoo deposition at different injection densities. (F) Example of fluorescence-based ion sensing from a dermal tattoo, illustrated here for Na^+^ detection. (G) Multiplex fluorescent/optical dermal tattoo pattern for simultaneous sensing of pH, Na^+^, and K^+^. Reproduced from Ref. [47], Jiang, N et al., Sensors and Actuators B: Chemical, 2020, 320. Elsevier. License No. 6291891357363. (For interpretation of the references to color in this figure legend, the reader is referred to the Web version of this article.)

The earliest and most influential example of this concept was provided by Ref. [3], which demonstrated minimally invasive dermal tattoo biosensors for colorimetric detection of pH, glucose, and albumin (Fig. 3-A and -B) in ex vivo porcine skin. In that study, the pH sensor responded across a broad range of 5.0–9.0 (Fig. 3-C), the glucose sensor detected concentrations up to 50.0 mmol L^−1^, and the albumin sensor quantitatively measured albumin up to 5.0 g L^−1^ with a reported limit of detection of 0.5 g L^−1^. The albumin response time was about 30 s, and the quantitative readout obtained by smartphone imaging yielded standard errors of 0.2 g L^−1^ in aqueous solution and 0.1 g L^−1^ in ex vivo tissue. Importantly, the three assays were also multiplexed into aesthetic tattoo-like patterns and quantified using a smartphone camera, demonstrating that dermal tattoos could serve as practical biochemical interfaces rather than merely decorative skin markings.

Building on this concept [44], advanced the field by introducing a segmented microneedle-based colorimetric dermal tattoo biosensor for multiplexed detection of pH, glucose, uric acid, and temperature (Fig. 3-D). The system responded to physiologically relevant ranges including pH 6.0–8.0, uric acid 0–1.5 mM, glucose 0–10 mM, and temperature 36–39 °C. The authors showed that pH differences as small as 0.5 units could be distinguished by the naked eye, while the microneedle-fabricated tattoo patterns achieved >85% fidelity and remained localized in the dermis for at least 30 days without infiltration into the epidermis. In vivo, the platform enabled simultaneous detection of four biomarkers, and pH and temperature monitoring remained reversible for at least 4 days, highlighting the feasibility of long-term multiplexed dermal tattoo sensing.

Together, these studies established colorimetric dermal tattoos as practical biochemical sensing platforms capable of both single-analyte and multiplexed monitoring directly within skin-associated fluid environments. At the same time, important limitations remain. Most current colorimetric dermal tattoo systems are still semi-quantitative, with performance influenced by ambient lighting, imaging angle, camera settings, and calibration procedures. In addition, the apparent visibility of the signal may vary with skin depth, tattoo density, and skin tone, while long-term chemical stability in vivo remains a key issue for translation. Nevertheless, colorimetric sensing laid the foundation for the field of dermal tattoo diagnostics and remains one of its most accessible and conceptually powerful branches.

### 3.2 Fluorescent and optical sensing

Following the initial success of colorimetric dermal tattoos, the field expanded into fluorescent and optical sensing**,** which offers higher sensitivity, better quantitative capability, and easier integration with portable readers and digital health systems. In these platforms, the implanted tattoo contains a fluorescent or photoresponsive material that changes its optical output in response to a biochemical analyte or an external physical stimulus. Compared with purely colorimetric systems, fluorescent and optical tattoos are especially attractive when low analyte concentrations, multiplexed detection, or smartphone-/device-assisted quantification are needed. They also preserve one of the core advantages of dermal placement: the sensing material remains in close contact with interstitial fluid while being shielded by the stratum corneum from sweat and environmental contamination.

A representative example is [47], which developed fluorescent dermal tattoo (Fig. 3-E) biosensors for electrolyte analysis. In this work, fluorescent sensors for H+, Na+ (Fig. 3-F), and K+ were tattooed into porcine skin and measured using a portable optical readout device composed of a smartphone, excitation LEDs, and optical bandpass filters. The platform provided quantitative detection of pH in the range 6.6–7.6**,** Na+ from 100 to 175 mmol L^−1^**,** and K+ from 2.0 to 6.0 mmol L^−1^, which are directly relevant to hydration assessment. Importantly, this system also demonstrated a multiplex capability by integrating separate fluorescent sensing regions for H+, Na+, and K+ within a single dermal tattoo platform, enabling simultaneous electrolyte analysis from the same skin site (Fig. 3-G).

**Fig. 4:**
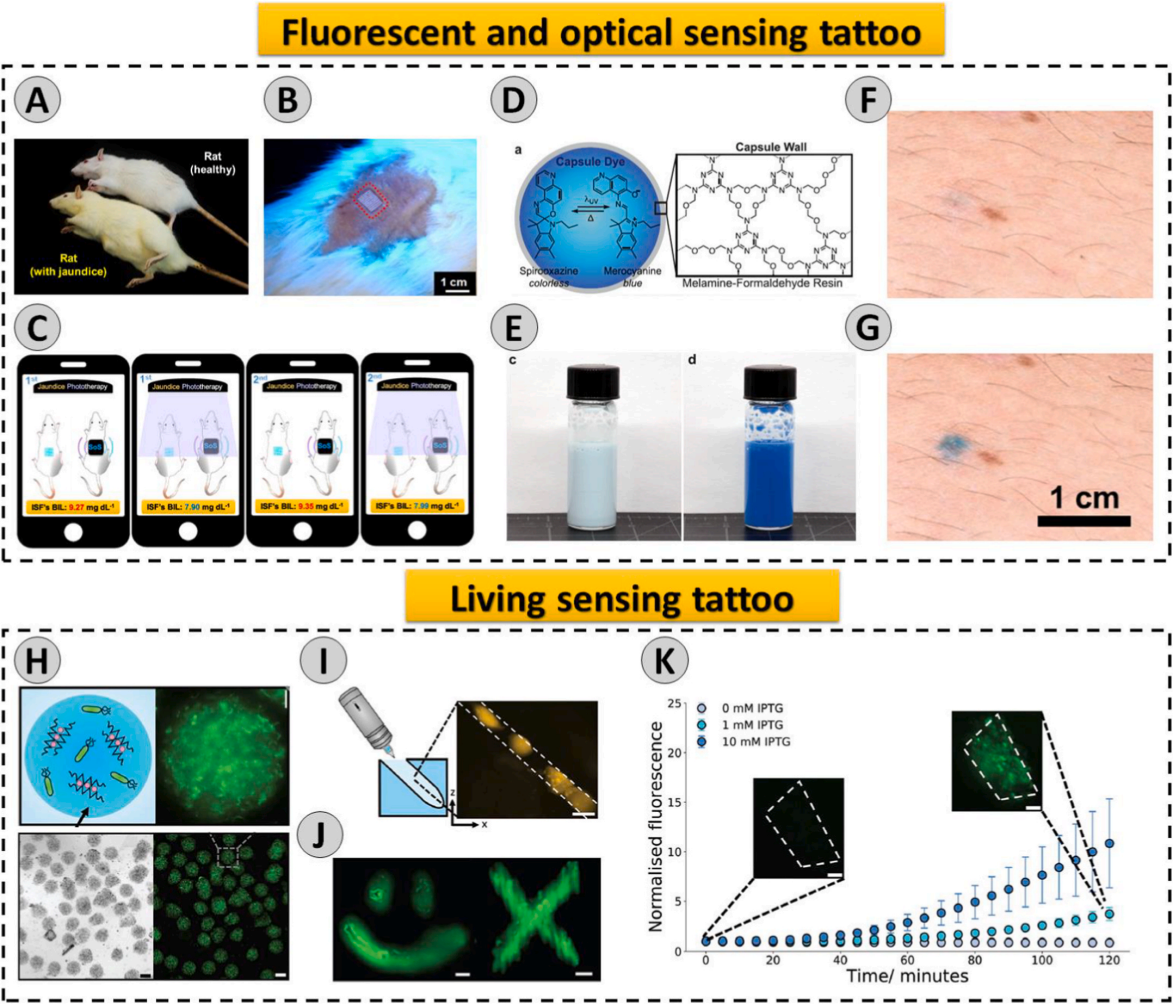
Representative examples of fluorescent/optical and living dermal tattoo sensing. (A) Rat model used for bilirubin sensing, comparing healthy and jaundiced animals. (B) Fluorescent dermal nanotattoo formed in skin for optical bilirubin monitoring. (C) Smartphone-based interface for wearable readout and digital tracking of bilirubin levels. Adapted with permission from Ref. [46], Tabatabaee, R. S.; et al., *ACS Nano*, 2024, 18(41). Copyright © 2024 American Chemical Society. (D) Structural design of the photochromic nanocapsule used for optical tattoo sensing. (E) Photochromic tattoo ink before and after light activation [48]. (F) Near-invisible photochromic tattoo in skin before UV exposure. (G) Visible activation of the photochromic tattoo after UV exposure. Adapted with permission from Ref. [48], Butterfield, J. L.; et al., *ACS Nano*, 2020, 14(10). Copyright © 2020 American Chemical Society. (H) Living dermal tattoo concept based on engineered bacteria encapsulated in alginate microgels, with representative microscopic images. (I) Tattoo-gun deposition of living microgels into a skin-mimicking substrate. (J) Examples of patterned fluorescent outputs generated by living tattoo biosensors. (K) Time-dependent fluorescence response of the living dermal tattoo to different IPTG concentrations, demonstrating stimulus-dependent sensing activity. Adapted from Ref. [45], Allen et al., Advanced Science, 2024. https://doi.org/10.1002/advs.202309509. Licensed under CC BY 4.0.

An even more advanced example is [46], which reported an invisible fluorescent dermal nanotattoo for bilirubin monitoring in neonatal jaundice (Fig. 4-A and -B). The tattoo was fabricated using GelMA dissolving/hydrogel microneedles loaded with highly fluorescent carbon quantum dots (CQDs) and was paired with a wearable optoelectronic reader containing a multispectral sensor, Bluetooth module, and smartphone application. The sensing mechanism relied on bilirubin-induced fluorescence quenching and selective signal recovery under blue-light exposure used in phototherapy (Fig. 4-C). Importantly, the system showed a very high correlation with blood bilirubin measurements in ex vivo and in vivo studies, while also supporting **c**ontinuous and reversible monitoring during phototherapy. The authors further emphasized its low cost, reporting an approximate cost of €0.17 per patch and €49 per reader, highlighting its translational potential as a smart point-of-care device.

A broader optical direction is illustrated by Ref. [48], which introduced “solar freckles,” a photochromic intradermal tattoo for real-time UV radiometry. Instead of detecting a biochemical analyte in ISF, this platform used nanoencapsulated leuco dyes (Fig. 4-D) that became progressively bluer (Fig. 4-E) with increasing UV irradiance (Fig. 4-F and -G). In ex vivo porcine skin, the tattoo response was quantified by ΔE*ab color change as a function of UVA/UVB intensity, and the recovery of the ground state followed first-order kinetics with k = 0.087 s^−1^. In a human pilot study, the tattoo reached ΔE*ab = 27 after activation by a 395 nm UV-LED lamp, while sunscreen application reduced tattoo visibility by about 70% **(**ΔE*ab = 19**)** after 4 weeks in vivo. Even after 8 months, the tattoo still retained detectable function (ΔE*ab = 8.8), indicating a service life on the order of months to years**,** far longer than most skin-adhered UV wearables.

Taken together, these studies show that fluorescent and optical dermal tattoos have substantially expanded the functional range of the field. They support biochemical monitoring, such as bilirubin and electrolyte sensing, but also environmental sensing, such as UV exposure. They are also more naturally compatible with portable optical hardware, smartphone quantification, and digital-health frameworks than purely colorimetric tattoos. At the same time, these systems introduce new challenges, including dependence on excitation sources or specialized readers, susceptibility to calibration and lighting effects, and potential issues such as photobleaching or long-term optical drift. Nevertheless, fluorescent and optical sensing clearly represent one of the most technologically sophisticated branches of dermal tattoo diagnostics.

### 3.3 Living biosensing

A particularly innovative but still very limited direction in dermal tattoo diagnostics is living biosensing, in which engineered biological systems themselves serve as the sensing element. The key example is [45], which introduced engineered *E. coli* encapsulated within alginate microgels (Fig. 4-H) and tattooed into an agarose skin mimic using a commercial tattoo gun (Fig. 4-I and -J). Importantly, this study was primarily a proof-of-concept rather than a demonstration of clinically established biomarker sensing: the bacteria were designed to respond to a model biochemical cue, IPTG, and a biophysical cue, temperature, rather than to a specific medical analyte. The bacteria-containing microgels had a mean diameter of 151 μm, making them suitable for tattoo delivery, and were engineered to respond to both stimuli through fluorescent GFP output. Quantitatively, the system showed dose-dependent fluorescence at 1 and 10 mM IPTG (Fig. 4-K), while the temperature-responsive bacteria exhibited stronger GFP expression at 30 °C. After tattooing into the agarose skin mimic, the bacteria remained functional and produced a detectable signal after 4 h, confirming that the sensing activity was preserved after transdermal deposition.

A major strength of this approach is the use of hydrogel microencapsulation, which helps localize the bacteria, preserve viability, and improve biosafety by reducing uncontrolled dispersion. However, this strategy also introduces important challenges, including biosafety, containment, genetic stability, long-term viability, and regulatory complexity. Thus, although living biosensing is currently represented by only a single dermal tattoo study, it remains one of the most conceptually transformative directions in the field, with future potential for programmable and sense-and-respond tattoo systems.

### 3.4 Electrophysiological monitoring

In addition to chemical and optical sensing, dermal tattoos have recently been explored as electrophysiological interfaces. The key example is the work of Wei et al. [4], which extended tattoo electrodes from an on-skin format to an actual under-skin dermal configuration. The system used a soft and conductive PEDOT:PSS-based polymer blend containing ethylene glycol and LiTFSI, known as PPEL. This material showed high conductivity of 5165 S cm^−1^, stretchability up to 56.9%, and a skin-contact impedance of 10.6 kΩ cm^2^ at 100 Hz in the on-skin tattoo-electrode configuration. These values indicate strong electrical and mechanical performance compared with many reported dry epidermal electrodes.

**Fig. 5:**
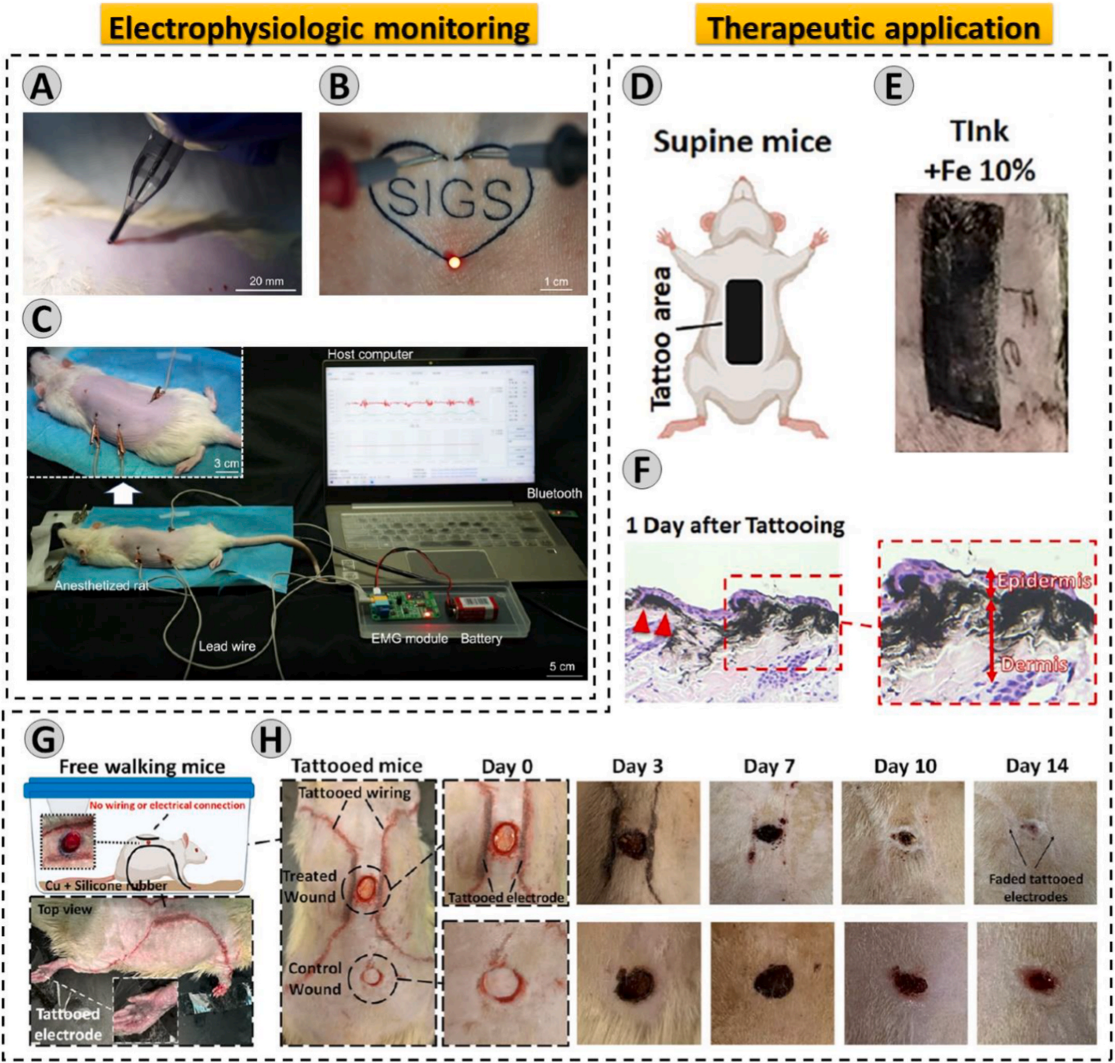
Electrophysiologic monitoring and therapeutic applications of dermal tattoos. (A) Dermal tattooing procedure for electrophysiological electrode formation. (B) Example of a conductive dermal tattoo pattern used as a bioelectronic interface. (C) In vivo electrophysiological monitoring setup using tattooed electrodes for EMG signal acquisition in a rat model. Adapted from Ref. [4], Wei et al., Ultraflexible Tattoo Electrodes for Epidermal and In Vivo Electrophysiological Recording, Cell Press, 2023. Copyright © Elsevier. (D) Schematic of the dorsal tattoo area used for therapeutic tattoo deposition in a supine mouse/rat model. (E) Representative image of a conductive therapeutic tattoo formulated with TInk + Fe (10%). (F) Histological section one day after tattooing, showing localization of tattoo pigments within the epidermal/dermal region. (G) Concept and experimental setup of a free-walking tattooed animal generating electrical output without external wiring. (H) Tattoo-assisted wound-healing model and representative wound images over time, showing the therapeutic effect of dermal tattoo electrodes and gradual fading of bioresorbable tattoo patterns. Adapted from Ref. [5], Shakibi et al., npj Flexible Electronics, 2025. https://doi.org/10.1038/s41528-025-00473-w. Licensed under CC BY-NC-ND.

Functionally, PPEL tattoo electrodes enabled ECG and EMG recording under static and dynamic conditions. In the on-skin configuration, the ECG signal-to-noise ratio was 25.3 dB, compared with 22.7 dB for commercial Ag/AgCl gel electrodes. More importantly, when the conductive ink was injected into rat skin as a dermal electrode (Fig. 5-A to -C), the dermal configuration produced higher in vivo EMG signal quality than epidermal electrodes, with an SNR of 13.8 dB compared with 10.2 dB. This improvement may be related to bypassing the highly resistive stratum corneum and achieving more direct electrical coupling with deeper tissue.

However, direct dermal-versus-epidermal impedance benchmarking remains limited. Therefore, current evidence supports improved signal quality and higher SNR, rather than a fully established claim of lower dermal impedance. Long-term electrical stability, signal drift, interconnection with external electronics, packaging, chronic biocompatibility, and standardized benchmarking remain important challenges before electrophysiological dermal tattoos can be considered mature bioelectronic interfaces.

## 4 Dermal tattoos for treatment and therapeutic applications

In contrast to the relatively broader literature on dermal tattoo-based sensing and diagnostics, therapeutic applications remain at a much earlier stage. To date, dermal tattoos have mainly been explored as diagnostic interfaces, whereas their use as active treatment platforms is still represented by very limited proof-of-concept evidence. Nevertheless, their intradermal localization, stable tissue contact, and mechanical conformity suggest that dermal tattoos could act as localized therapeutic conduits rather than only passive sensing elements. However, compared with sensing applications, therapeutic dermal tattoos require more rigorous evaluation of long-term safety, controllability, electrical dose delivery, degradation behavior, and translational feasibility.

At present, the clearest example of this direction is provided by Ref. [5], which introduced a dermal tattoo-based triboelectric nanogenerator system (Tat-TENG) for self-powered wound healing. In this platform, the skin served as the triboelectric layer, while conductive tattoo inks deposited in the dermis (Fig. 5-D to -F) functioned as the charge-transfer pathway. This configuration reduced the need for conventional external wires or adhesive surface electrodes and enabled body-motion-generated electrical stimulation to be delivered to the wound site (Fig. 5-G). In a small-animal wound model, treated animals were able to move freely while the system generated electrical output, and the Tat-TENG group showed approximately 80% wound closure compared with about 60% in the control group (Fig. 5-H). The study also introduced a glycerol-based Zn-containing conductive ink that gradually faded from the skin, suggesting the possibility of matching tattoo lifetime with the therapeutic window.

Despite these promising results, this therapeutic direction remains highly preliminary. The available evidence is limited to a single early-stage study, and several important questions remain unresolved. These include the reproducibility of electrical stimulation under variable body motion, control over stimulation dose and field distribution, long-term tissue response to conductive inks and nanoparticles, degradation kinetics of bioresorbable formulations, and the lack of standardized therapeutic benchmarks. Therefore, Tat-TENG should be viewed not as evidence of clinical readiness, but as an initial demonstration that dermal tattoos may be engineered as active, localized, and potentially transient bioelectronic therapeutic interfaces.

## 5 Fabrication strategies, materials and readout mechanisms

The performance of dermal tattoo biomedical systems is determined not only by their intended application, but also by the materials used, the method of deposition into the dermis, and the strategy by which the resulting signal is read and interpreted. These three elements are tightly interconnected: the fabrication approach influences the spatial distribution and stability of the tattoo, the functional material defines the sensing or therapeutic mechanism, and the readout method determines how effectively the platform can be translated into practical biomedical use. Accordingly, understanding dermal tattoos as biomedical interfaces requires a closer examination of their enabling technological components. This section therefore discusses the principal fabrication strategies, material classes, and readout mechanisms that currently define the design space of dermal tattoo systems.

### 5.1 Fabrication strategies for dermal tattoo platforms

The fabrication strategy of a dermal tattoo platform is a defining factor in its biomedical performance because it governs the depth of delivery, spatial distribution, reproducibility, invasiveness, and final functionality of the implanted material. In the current dermal tattoo literature, two main fabrication approaches have been used: microneedle-based delivery and conventional tattoo-gun deposition. Although both methods aim to place functional materials within the dermis, they differ substantially in terms of precision, ease of multiplexing, and compatibility with different material classes.

#### 5.1.1 Microneedle-based dermal tattooing

Microneedle-based strategies represent one of the most controlled and minimally invasive approaches for the fabrication of dermal tattoo platforms. In these systems, arrays of micron-scale needles are used to penetrate the epidermis and deliver sensing reagents into the upper dermis with relatively high precision in both depth and pattern geometry. This approach is particularly attractive for biomedical dermal tattoos because it combines repeatable insertion, reduced pain, and compatibility with multiplexed designs.

**Fig. 6:**
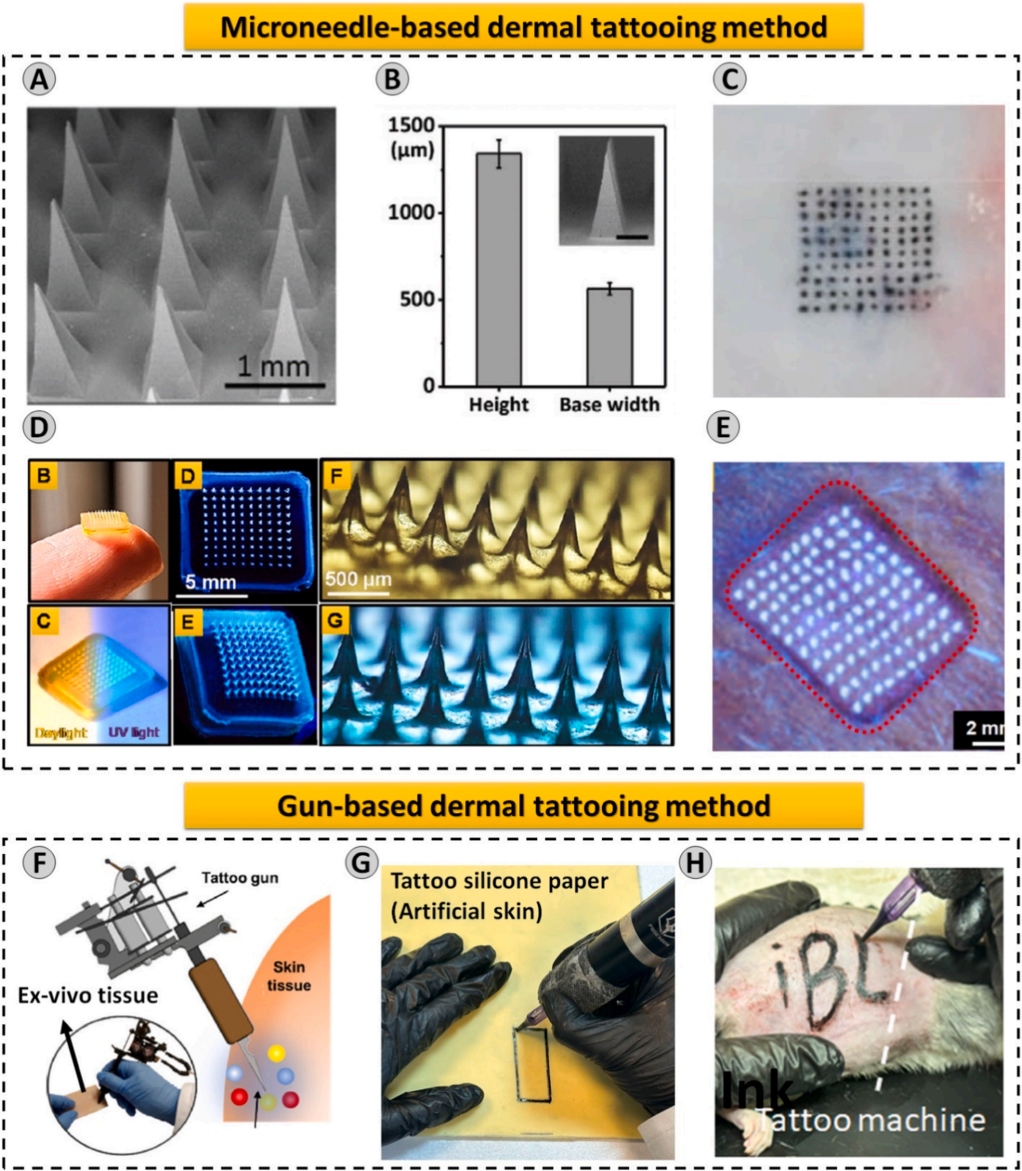
Representative fabrication strategies used for dermal tattoo platforms. (A–E) Representative examples of microneedle-based dermal tattooing, including microneedle morphology, dimensions, patterned tattoo formation, and dermal deposition in skin [44,46]. (F–H) Representative examples of gun-based dermal tattooing, showing tattoo-gun-assisted deposition of sensing or conductive inks in ex vivo tissue, artificial skin, and in vivo animal skin [5,47].

In [44], a four-area segmented hyaluronic-acid microneedle patch (Fig. 6-A and -B) was used to create a colorimetric dermal tattoo (Fig. 6-C) for multiplexed monitoring of pH, glucose, uric acid, and temperature. The microneedles dissolved after insertion, releasing the sensing reagents into the dermis and forming clearly defined tattooed regions suitable for visual and camera-based readout. One of the main advantages emphasized in this study was the improved consistency of penetration depth and angle compared with handheld tattooing methods, which is particularly important for uniform color development and reliable semi-quantitative sensing. Likewise [46], employed dissolving/hydrogel microneedles based on GelMA to fabricate an invisible fluorescent dermal nanotattoo (Fig. 6-D) excited by UV light (Fig. 6-E) for bilirubin sensing. In this case, microneedles also enabled minimally invasive deposition of fluorescent carbon quantum dots into the dermis for continuous monitoring.

Taken together, these studies show that microneedle-based fabrication is especially well suited for diagnostic dermal tattoos, particularly when precise reagent placement, painless application, and multiplexing are required. However, this approach also has limitations. The material payload that can be loaded into microneedles is restricted, the fabrication process can be more elaborate than conventional tattooing, and the method is more naturally compatible with sensing reagents than with some high-load conductive formulations used in bioelectronic systems.

#### 5.1.2 Tattoo-gun-based dermal deposition

The second and more widely used fabrication route in the current field is conventional tattoo-gun deposition (Fig. 6-F to -H). This approach directly builds on traditional tattooing practice: a rapidly oscillating needle repeatedly penetrates the skin and delivers ink or functional material into the dermis. In the reviewed papers, tattoo-gun-based deposition was used for living biosensing [45], fluorescent electrolyte sensing [47]**,** photochromic UV sensing [48]**,** colorimetric metabolite sensing [3]**,** dermal electrophysiological electrodes [4]**,** and therapeutic conductive tattoos [5]**.** This makes the tattoo gun the most broadly adopted fabrication tool across the current dermal tattoo literature.

A major advantage of tattoo-gun deposition is its versatility. It can accommodate a wide range of functional materials, including dyes, fluorescent probes, microgels, nanoparticles, and conductive inks, and it also allows flexible geometric patterning (Fig. 6-H) that can be aligned with decorative tattoo practice. In addition, it is an accessible and already well-established method outside biomedicine, which may ultimately support translational scalability. Several papers also noted that tattooing parameters such as depth, angle, and density strongly influence material distribution in the dermis and therefore affect signal visibility, localization, and functional performance. In Ref. [3], for example, tattoo density, position, depth, and angle were systematically characterized to improve biosensor injection. In Ref. [45], the tattoo gun allowed the microgel “ink” to be positioned across different depths and orientations in the skin mimic.

At the same time, tattoo-gun deposition introduces more variability than microneedle-based methods. Because the final distribution of material depends on operator handling, needle angle, penetration depth, and ink rheology, reproducibility can be more difficult to control. These factors become especially important in biomedical systems where quantitative readout is required. Nevertheless, the current literature shows that conventional tattoo-gun deposition is the dominant fabrication strategy for dermal tattoo platforms and is uniquely capable of supporting not only sensing but also bioelectronic and therapeutic applications.

Taken together, the present dermal tattoo literature supports a simple but important conclusion: microneedles and tattoo guns are the two principal fabrication strategies currently defining the field. Microneedle-based approaches provide greater control and are especially well suited to multiplexed sensing platforms, while tattoo-gun deposition is more versatile and has been used across nearly all other dermal tattoo applications, including optical, living, electrical, and therapeutic systems. Thus, fabrication choice should be viewed not merely as a procedural detail, but as a central design parameter that shapes the performance, reproducibility, and translational potential of dermal tattoo biomedicine.

Fabrication parameters directly influence dermal tattoo performance and should be considered as design variables rather than only delivery methods. In tattoo-gun-based systems, needle depth, injection angle, tattoo density, ink spreading, and operator variability affect the final position and distribution of functional materials in the dermis, which can alter optical visibility, signal intensity, electrical coupling, and reproducibility [3,47]. Microneedle-based systems partially address these limitations by providing more controlled insertion geometry and patterned reagent delivery, as shown in colorimetric and bilirubin-sensing dermal tattoos [44,46]. In living tattoo systems, delivery compatibility also depends on microgel size and mechanical stability, because the microgels must pass through the tattoo needle while retaining bacteria and allowing analyte diffusion [45]. Therefore, future studies should report fabrication parameters such as delivery depth, pattern fidelity, material spreading, delivered dose, and inter-operator variability.

### 5.2 Functional materials in dermal tattoo systems

The functional materials embedded within the dermis are the core determinants of dermal tattoo performance, defining whether the platform acts as a colorimetric sensor, fluorescent probe, living biosensor, electrical interface, or therapeutic conduit. Unlike conventional decorative tattoos, biomedical dermal tattoos rely on materials that interact actively with analytes, light, electrical signals, or surrounding tissue. Accordingly, the choice of material influences not only the sensing or therapeutic mechanism, but also the biocompatibility, stability, reversibility, long-term persistence, and clinical translatability of the platform. The current literature reveals a diverse but still emerging materials landscape, spanning classical colorimetric reagents, fluorescent and photoresponsive nanomaterials, engineered living cells, and conductive or bioresorbable bioelectronic inks.

#### 5.2.1 Colorimetric sensing chemistries

Colorimetric materials were among the earliest and most accessible functional chemistries used in dermal tattoo biosensing because they convert biochemical changes directly into visible color variations. In dermal tattoos, these systems are especially attractive because the sensing material is placed beneath the stratum corneum, allowing interaction with interstitial fluid while being protected from surface contamination. However, the actual “tattoo ink” in these systems is not a conventional decorative pigment alone; rather, it is a functional formulation composed of colorimetric reagents embedded in an appropriate carrier or delivery matrix**.**

**Fig. 7:**
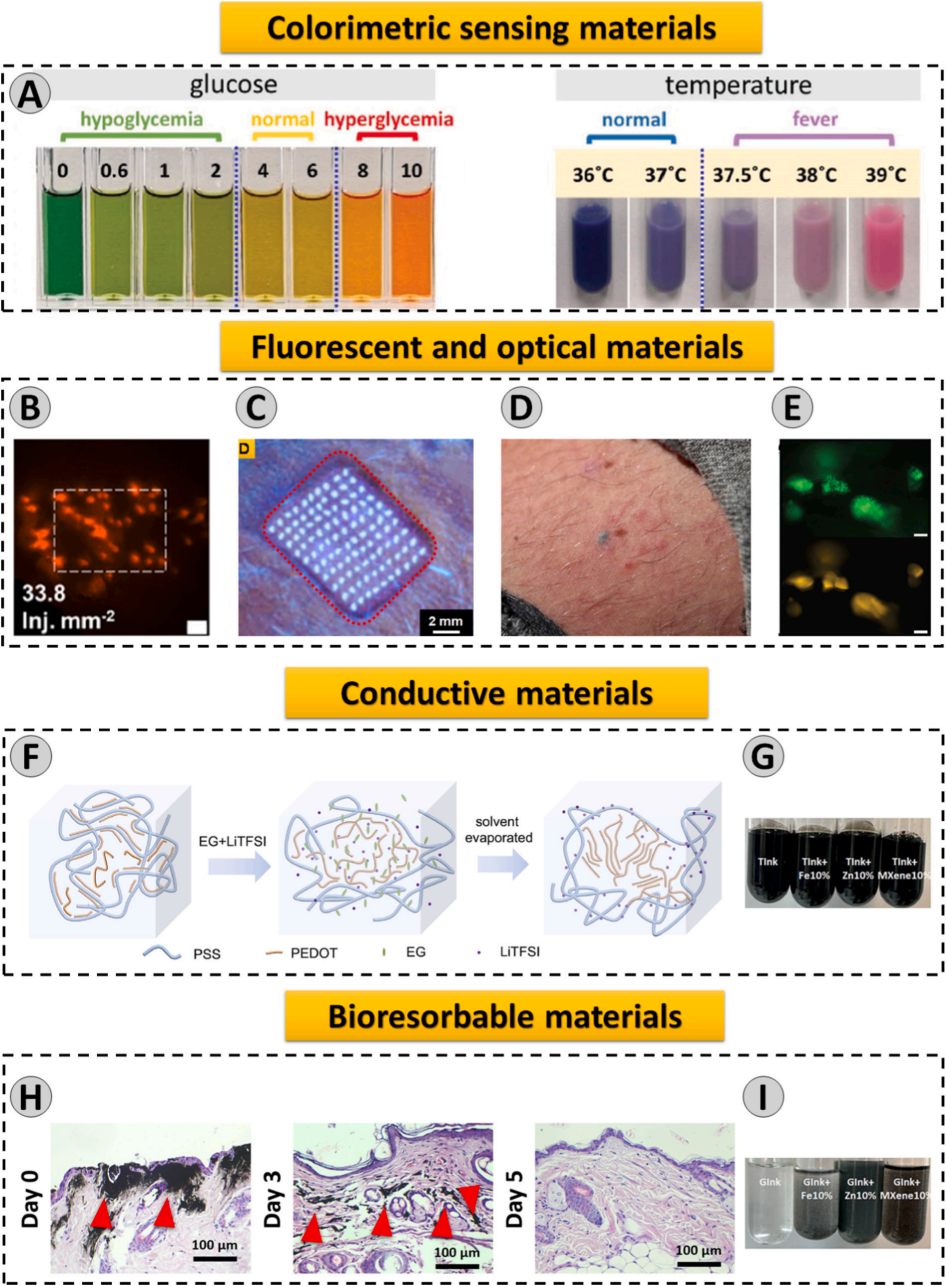
Representative material classes used in dermal tattoo systems. (A) Examples of colorimetric sensing materials, showing analyte-responsive color changes for glucose and temperature [44]. (B–E) Examples of fluorescent and optical materials, including fluorescent tattoo deposition, microneedle-based fluorescent patterns, photochromic optical tattoos in skin, and fluorescence-generating living microgels [[45], [46], [47], [48]]. (F–G) Examples of conductive materials, including the PEDOT:PSS/EG/LiTFSI conductive network and nanoparticle-enhanced tattoo inks [4,5]. (H–I) Examples of bioresorbable materials, showing the gradual disappearance of tattoo material in histological sections over time and representative bioresorbable ink formulations. Readout and signal interpretation mechanisms [5]. (For interpretation of the references to color in this figure legend, the reader is referred to the Web version of this article.)

In [3], the dermal tattoo was created using a commercial tattoo gun to inject colorimetric biosensor formulations (Fig. 7-A) directly into ex vivo porcine skin. The active sensing chemistries were: methyl red, bromothymol blue, and phenolphthalein for pH sensing; **g**lucose oxidase, peroxidase, and 3,3′,5,5′-tetramethylbenzidine (TMB) for glucose sensing; and 3′,3″,5′,5″-tetrachlorophenol-3,4,5,6-tetrabromosulfophthalein for albumin sensing. Thus, in this work, the “ink” was essentially a set of chromogenic molecular reagents and enzyme-based sensing cocktails delivered into the dermis by tattooing.

In [44], the strategy was different because the sensing materials were not delivered by a tattoo gun but by hyaluronic-acid microneedle patches. Here, the colorimetric tattoo was formed when the dissolving HA microneedles released their payload into the dermis. The sensing formulations included thymol blue, methyl red, and phenolphthalein for pH detection; glucose oxidase for glucose sensing; TMB together with uricase and horseradish peroxidase for uric acid sensing; and a temperature-responsive powder for thermal sensing. In this case, the effective tattoo material was therefore a combination of HA as the delivery matrix and encapsulated colorimetric/enzymatic reagents as the active functional components.

Taken together, these studies show that colorimetric dermal tattoo systems rely on two essential material components: (i) the active sensing chemistry, usually based on dyes or enzyme-coupled chromogenic reactions, and (ii) the carrier platform that introduces those materials into the dermis, such as direct tattoo-gun injection or dissolving HA microneedles. Their main strengths are simplicity, visual interpretability, and compatibility with smartphone-based readout, although their performance remains sensitive to calibration, lighting conditions, and the long-term stability of the colorimetric reagents in the dermal environment.

#### 5.2.2 Fluorescent and optical materials

Fluorescent and optical materials have significantly broadened the capabilities of dermal tattoo systems by enabling higher sensitivity, multiplexing potential, and compatibility with portable optical readers. In contrast to colorimetric systems, which rely on visible color changes, fluorescent dermal tattoos produce optical signals that can often be measured more quantitatively and with greater analytical precision. As in the colorimetric case, these systems are not based on decorative tattoo pigments alone; rather, the “tattoo ink” consists of optically active functional materials delivered into the dermis through an appropriate carrier platform, such as microneedles, tattoo-gun deposition, or hydrogel microgels.

Several papers in the current field fall into this category. In Ref. [47] (Fig. 7-B), the tattoo material consisted of fluorescent ion-sensitive probes for H+, Na+, and K+, enabling hydration-related monitoring through a portable optical reader and smartphone analysis. In Ref. [46] (Fig. 7-C), the optical tattoo was formed using fluorescent carbon quantum dots (CQDs) loaded into GelMA dissolving/hydrogel microneedles, creating an invisible fluorescent dermal nanotattoo for bilirubin detection. [48], while not fluorescence-based in the strict sense, also belongs to the broader optical category because it used photochromic nanocapsules containing leuco dyes as the tattoo material, producing visible UV-dependent color shifts for intradermal UV radiometry (Fig. 7-D). In addition [45], can also be considered part of this group because its signal output was fluorescent, although the sensing system was biologically distinct: here, the tattoo material consisted of engineered *E. coli* encapsulated in alginate microgels**,** which generated a fluorescent GFP signal (Fig. 7-E) in response to temperature or IPTG stimulation. Thus [45], shares the same general optical-output logic as other fluorescent tattoos, but with a living biosensing system as the active material.

This family of materials offers several advantages. Optical materials can support greater analytical sensitivity, enable multiplexing across wavelengths or emission channels, and integrate naturally with smartphone cameras or wearable readers. They also allow more sophisticated digital-health architectures, especially when combined with optical filtering, excitation sources, and app-based quantification. However, they may require external instrumentation, careful calibration, and compensation for environmental light conditions. Some fluorescent materials may also face issues such as photobleaching, signal drift, or long-term optical instability in vivo. Despite these challenges, fluorescent and optical materials represent one of the most technologically advanced branches of dermal tattoo sensing.

#### 5.2.3 Conductive and bioelectronic inks

Conductive materials form the basis of dermal tattoo systems intended for electrical recording, stimulation, or self-powered operation. In contrast to optical and colorimetric tattoos, these platforms depend on materials that can establish efficient charge transport within or beneath the skin. This category includes conducting polymers, nanoparticle-loaded tattoo inks, and hybrid conductive formulations designed to bridge soft tissue and electronic function.

Article [4] represents a major advance in this area, using a highly conductive polymer blend based on PEDOT:PSS, ethylene glycol (EG), and LiTFSI (Fig. 7-F) to create ultraflexible tattoo electrodes for epidermal and dermal electrophysiological recording. Research done by Ref. [5] expanded conductive dermal inks toward therapeutic bioelectronics by integrating conductive nanoparticles such as Fe, MXene, and Zn into tattoo-compatible ink systems (Fig. 7-G). In that work, commercial tattoo ink and a glycerol-based bioresorbable ink acted as carriers for conductive fillers, allowing charge transfer in a triboelectric treatment system.

Conductive and bioelectronic inks are highly attractive because they open dermal tattoos to applications beyond chemical sensing, including signal acquisition, electrical communication with tissue, stimulation, and self-powered therapy. However, their development involves several design trade-offs. High conductivity must be balanced with mechanical softness, stability in biological environments, acceptable rheology for tattooing or printing, and minimal toxicity. Nanoparticle sedimentation, long-term material persistence, and tissue compatibility are especially important when these materials are intended for under-skin placement. Even so, conductive tattoo materials are central to the transition of dermal tattoos from sensors to active bioelectronic platforms.

#### 5.2.4 Transient and bioresorbable materials

A particularly promising direction in dermal tattoo technology is the use of transient or bioresorbable functional materials, which allow the platform to disappear naturally after completing its diagnostic or therapeutic task. This concept is especially valuable in biomedical applications where permanent implantation is unnecessary or undesirable, such as short-term wound care, post-operative monitoring, or temporary treatment.

The clearest example in the current literature is [5], which introduced a glycerol-based bioresorbable ink named as Gink (Fig. 7-I) loaded with Zn nanoparticles for transient tattoo-based triboelectric therapy. In that system, the tattoo function gradually vanished as the ink faded from the skin (Fig. 7-H), synchronizing the disappearance of the device with the healing process. This approach illustrates how material transience can be built directly into the therapeutic design of dermal tattoos.

Bioresorbable materials offer several important benefits: they eliminate the need for device removal, reduce long-term foreign-body burden, and align naturally with temporary medical needs. At the same time, they introduce design challenges related to degradation rate, retention of functionality during the intended lifetime, storage stability, and predictable in vivo behavior. Although still underexplored, transient materials may become increasingly important as dermal tattoos move toward clinical translation and personalized therapeutic use.

The reported studies also provide partial information for building a materials-design framework for dermal tattoos. Delivery compatibility has been most clearly addressed: He et al. [44] used microneedle patches to achieve controlled dermal delivery of colorimetric reagents, while Yetisen et al. [3], Jiang et al. [47], Allen et al. [45], Wei et al. [4], and Shakibi et al. [5] demonstrated tattoo-gun-based delivery of colorimetric probes, fluorescent probes, living microgels, conductive inks, and therapeutic conductive formulations, respectively. Retention and localization have also been partially demonstrated. For example, the microneedle colorimetric tattoo remained localized in the dermis for at least 30 days [44], photochromic “solar freckle” tattoos retained detectable UV-responsive function for months in human skin [48], and injected dermal electrodes maintained electrical function for a limited period in vivo [4]. Stability and reversibility were also system-dependent: pH and temperature responses were reversible for several days in the microneedle platform [44], the bilirubin nanotattoo supported reversible optical monitoring during phototherapy [46], and the photochromic UV tattoo showed reversible color changes after UV exposure and removal [48]. Degradation has been considered mainly in dissolving microneedle systems [44,46] and in the bioresorbable Zn-containing therapeutic tattoo ink that gradually faded during wound healing [5].

However, several important material parameters remain insufficiently studied across the current dermal tattoo literature. Quantitative permeability and analyte-diffusion coefficients are rarely reported, although alginate microgels in the living tattoo study were designed to allow small-molecule diffusion while retaining engineered bacteria [45]. Leaching, extractables, long-term migration, and clearance of functional materials have not been systematically evaluated, except indirectly through observations of dermal localization or pattern retention. Similarly, ink rheology, viscosity, shear-thinning behavior, sedimentation of nanoparticles, and injectability are generally not quantified, even though they are likely to strongly affect tattoo-gun delivery and spatial distribution in the dermis. Sterilization compatibility is also largely unaddressed, particularly for functional dyes, nanomaterials, hydrogels, conductive polymers, and living systems. Therefore, future dermal tattoo studies should report not only sensing or therapeutic performance, but also material permeability, retention, leaching, stability, reversibility, degradation products, rheology, sterilization compatibility, and delivery reproducibility as key parameters for translation.

A clearer biosensor performance framework is needed to compare dermal tattoo sensing platforms. Current studies mainly report dynamic range and selected analytical metrics. Yetisen et al. reported sensing of pH 5.0–9.0, glucose up to 50.0 mM, and albumin up to 5.0 g L^−1^, with an albumin LOD of 0.5 g L^−1^ and response time of about 30 s [3]. He et al. [44] reported pH 6.0–8.0, glucose 0–10 mM, uric acid 0–1.5 mM, and temperature 36–39 °C. Jiang et al. [47] reported pH 6.6–7.6, Na^+^ 100–175 mM, and K^+^ 2.0–6.0 mM, while Tabatabaee et al. [46] reported bilirubin detection over 3–20 mg dL^−1^ with an LOD of 0.5 mg dL^−1^. However, sensitivity, selectivity, hysteresis, drift, calibration stability, and clinical validation remain insufficiently reported. Future studies should therefore use standardized metrics for performance benchmarking.

### 5.3 Read-out mechanisms

The readout mechanism is a defining component of any dermal tattoo biomedical platform because it determines how the biological or physical information embedded within the tattoo is translated into a clinically meaningful signal. Even when the implanted sensing or conductive material performs effectively in the dermis, the utility of the platform ultimately depends on whether the output can be interpreted simply, accurately, reproducibly, and, ideally, in real time. Across the current literature, dermal tattoo systems rely on four main classes of readout: visual readout, optical reader-based detection, smartphone-assisted quantification, and electrical readout. These strategies differ substantially in complexity, cost, sensitivity, and suitability for decentralized healthcare.

#### 5.3.1 Visual and naked-eye readout

Visual readout is the simplest and most intuitive signal interpretation strategy used in dermal tattoo systems. In this mode, the tattoo undergoes a directly observable color change in response to a target analyte or external stimulus, allowing the user to interpret the result without the need for specialized instrumentation. This approach is particularly attractive for point-of-care and self-monitoring applications because it minimizes device dependence and can support fast, user-friendly decision-making.

**Fig. 8:**
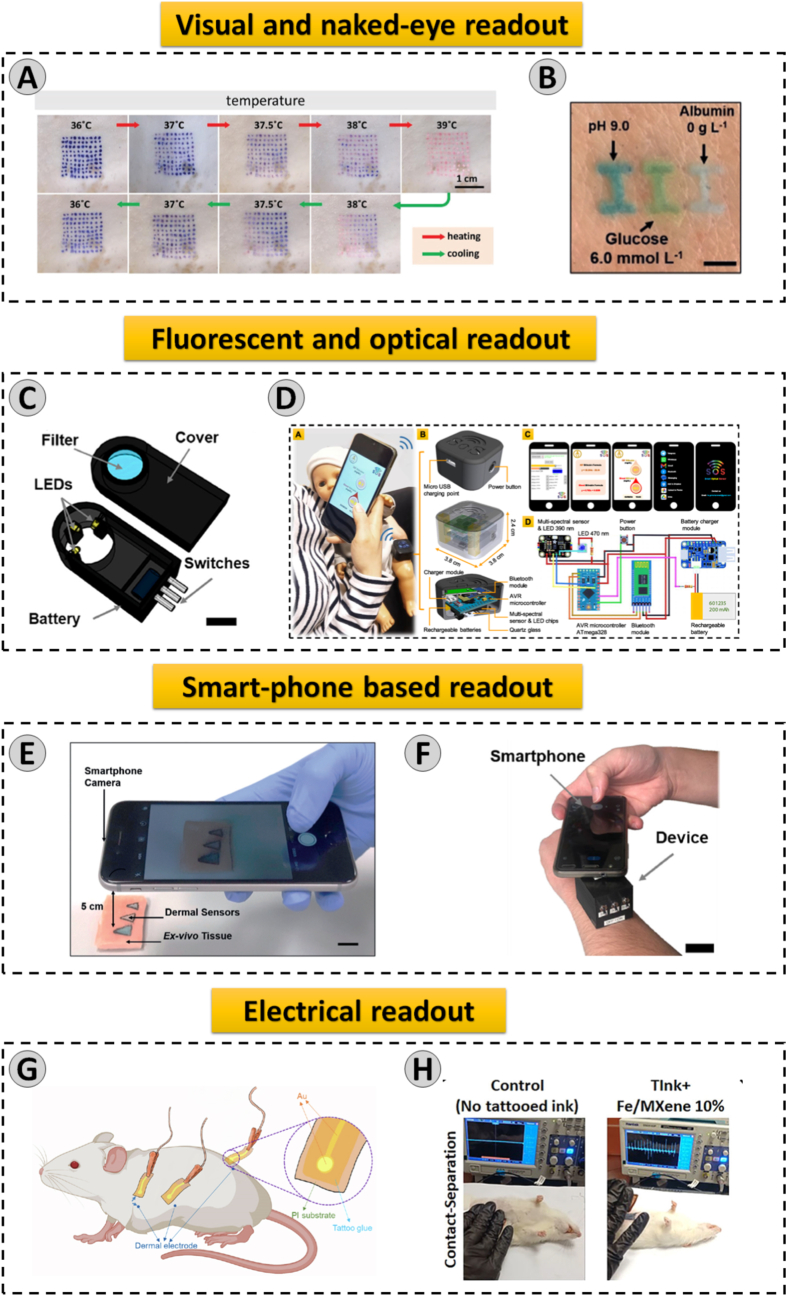
Representative readout strategies used in dermal tattoo systems. (A–B) Examples of visual and naked-eye readout, showing direct interpretation of colorimetric tattoo responses for temperature, pH, glucose, and albumin [3,44]. (C–D) Examples of fluorescent and optical readout, including a portable optical reader and a wearable optoelectronic system for fluorescence-based dermal tattoo sensing [46,47]. (E–F) Examples of smartphone-based readout, showing camera-assisted imaging and portable smartphone-coupled detection of dermal tattoo signals [3,47]. (G–H) Examples of electrical readout, including electrophysiological monitoring with dermal electrodes and triboelectric signal generation from conductive tattooed skin [4,5].

Colorimetric dermal tattoo systems in Refs. [3,44] strongly rely on this principle, where changes in biomarker concentration are translated into visible color changes within the tattooed sensing regions (Fig. 8-A and -B). Research article [48] also follows this general logic through photochromic intradermal UV tattoos that provide direct visual feedback regarding ultraviolet exposure. The main strength of visual readout is its accessibility: it is low-cost, immediate, and easy to use. For this reason, it aligns well with the original promise of dermal tattoos as minimally invasive yet user-centered biomedical interfaces.

However, visual interpretation also has important limitations. It is often subjective, influenced by user perception, ambient illumination, skin tone, imaging angle, and the depth or density of tattoo deposition. As a result, purely visual readout is often best suited for qualitative or semi-quantitative assessment, rather than highly precise measurement. Nevertheless, as an instrument-free strategy, it remains one of the most attractive features of dermal tattoo biosensors.

#### 5.3.2 Optical reader-based systems

To improve analytical precision beyond naked-eye observation, several studies have integrated dermal tattoos with dedicated optical readout systems. In these platforms, the tattoo responds through fluorescence or another optical change, which is then captured by an external reader containing excitation sources, filters, and photodetectors or spectrometric components. This strategy is particularly useful when the sensing signal is subtle, wavelength-specific, or intended for quantitative analysis [47]. used a portable optical device composed of a smartphone, excitation LEDs, and optical filters to detect fluorescence changes from dermal tattoo electrolyte sensors (Fig. 8-C), while [46] further advanced this concept by coupling a fluorescent bilirubin nanotattoo with a wearable optoelectronic reader incorporating a multispectral sensor, microcontroller, Bluetooth module, and smartphone application (Fig. 8-D). In addition [45], relied on fluorescence microscopy and timelapse microscopy to monitor the GFP output of engineered bacteria encapsulated in tattooed alginate microgels, showing that microscopic optical imaging can also serve as an effective readout strategy for living dermal tattoo systems. Together, these studies show that optical reader-based systems can transform dermal tattoos into more robust diagnostic platforms by improving signal sensitivity and reducing reliance on subjective visual interpretation.

#### 5.3.3 Smartphone-based quantification

Smartphones occupy a unique position in dermal tattoo readout because they bridge simple imaging and digital health infrastructure. In many dermal tattoo systems, the smartphone functions either as a camera-based quantification tool or as the central node in a broader sensing and connectivity ecosystem. This approach is particularly attractive because smartphones are already widely available, portable, and equipped with imaging, computation, and communication capabilities.

Published results in Ref. [44] indicate using camera-based analysis to achieve semi-quantitative measurement of colorimetric changes in a multiplexed dermal tattoo biosensor. Similarly [3], employed smartphone imaging for quantitative interpretation of metabolite-sensitive tattoos (Fig. 8-E). In Ref. [47], a smartphone application converted fluorescence signals into electrolyte concentrations (Fig. 8-F), while [46] pushed this strategy toward smart diagnostics by integrating the tattoo sensor with a wearable reader and app-based communication framework.

Smartphone-based quantification offers several important advantages. It increases accessibility, facilitates semi-quantitative or quantitative analysis, enables data storage and transmission, and supports remote or longitudinal monitoring. It is also highly compatible with point-of-care and home-based use. However, this strategy is not without limitations. Readout accuracy can be affected by camera variability, lighting conditions, image calibration, and the need for robust compensation algorithms. Even so, smartphones are likely to remain one of the most practical and scalable readout tools for future dermal tattoo systems.

#### 5.3.4 Electrical readout

Electrical readout is the dominant mechanism in dermal tattoo systems designed for electrophysiological monitoring or therapeutic bioelectronics. Instead of producing a visible or optical response, these platforms generate, transmit, or record electrical signals directly. This makes them particularly important for applications involving bioelectronic interfacing, physiological signal acquisition, energy harvesting, and electrical stimulation.

Paper [4] is a clear example of electrical readout in a sensing context, where dermal tattoo electrodes were used to record electrophysiological signals such as ECG and EMG with high precision (Fig. 8-G). In this case, the dermal interface increased signal to noise ratio (SNR) and quality by reducing motion artifacts and improving electrical communication with tissue. Paper [5] represents the therapeutic counterpart, in which conductive dermal tattoos functioned as a charge-transfer pathway in a triboelectric nanogenerator system (Fig. 8-H), enabling self-powered wound healing through electrically mediated stimulation.

Electrical readout offers several major strengths: it supports real-time operation, high temporal resolution, and direct integration with bioelectronic devices. It is essential for applications where the goal is not only to sense but also to communicate electrically with tissue or deliver therapy. However, electrical systems typically require associated electronics, noise control, stable conductive interfaces, and more elaborate instrumentation than purely visual or optical tattoos. As dermal tattoo technology evolves toward treatment and closed-loop systems, electrical readout will likely become increasingly important.

Readout strategy also determines both the strength and limitations of each platform. Visual and smartphone-based colorimetric readouts are low-cost and simple, but are sensitive to ambient light, camera settings, skin tone, tattoo depth, and background pigmentation [3,44]. Fluorescent and optical readouts improve sensitivity and quantitative analysis, but require excitation sources, filters, calibration, and correction for photobleaching, tissue scattering, melanin, and hemoglobin absorption [46,48]. Electrical readouts provide high temporal resolution for electrophysiological monitoring, but are affected by interfacial noise, motion artifacts, connection stability, tissue remodeling, and packaging [4]. Triboelectric readouts can support self-powered operation, but their output depends strongly on body motion, contact pressure, material pairing, and mechanical consistency [5]. Thus, fabrication and readout should be evaluated together, because the final performance of dermal tattoos depends on both material placement and signal acquisition conditions.

Overall, dermal tattoo systems combine a relatively limited number of fabrication routes, mainly microneedle-based delivery and tattoo-gun deposition, with a diverse range of functional materials and readout mechanisms. This combination enables applications ranging from biochemical sensing and electrophysiological monitoring to environmental exposure tracking and therapy. As summarized in Table 2, the performance of dermal tattoo platforms is determined not only by the implantation strategy, but also by the interaction between fabrication method, material chemistry, signal transduction mechanism, and intended biomedical function. Different material systems enable distinct capabilities, including visual metabolite sensing, selective electrolyte detection, stable optical readout, high-quality bioelectrical recording, and self-powered therapeutic operation. Therefore, material selection plays a central role in defining sensitivity, selectivity, signal stability, biocompatibility, and long-term usability.

**Table 2:** Summary of the reported dermal tattoo biomedical platforms, including their main function, model system, tattooing mechanism, functional materials, readout strategy, measurement range, and biomedical application.

Ref.	Main function	Animal/skin model	Tattooing mechanism	Tattoo ink/functional material	Readout mechanism	Range of measurement	Biomedical application
[44]	Multiplexed sensing	Rabbit skin ex vivo and in vivo	Microneedle patch	HA microneedles loaded with thymol blue, methyl red, phenolphthalein, glucose oxidase, TMB, uricase, HRP, and temperature-responsive powder	Visual and camera	pH 6.0–8.0; glucose 0–10 mM; uric acid 0–1.5 mM; temperature 36–39 °C	Multiplexed health-related biomarker monitoring
[45]	Living sensing	Agarose skin mimic; porcine skin feasibility study	Tattoo gun	Alginate microgels containing engineered *E. coli*	Fluorescent/optical (including fluorescence microscopy)	IPTG 1 and 10 mM; temperature response around 30 °C	Living dermal tattoo biosensing for biochemical and biophysical cues
[46]	Fluorescent sensing	Rat skin in vivo; human skin; ex vivo/in vivo ISF context	Microneedle	GelMA dissolving/hydrogel microneedles containing carbon quantum dots (CQDs)	Optical, wearable optoelectronic reader, smartphone/IoT	Bilirubin monitoring; high correlation with blood bilirubin	Bilirubin monitoring for neonatal jaundice and therapeutic monitoring
[47]	Multiplexed sensing	Porcine skin ex vivo	Tattoo gun	Fluorescent ion-sensitive probes for H+, Na+, and K+ (seminaphthorhodafluor, diaza-crown ethers), with tattoo-compatible formulation	Optical + smartphone-based	pH 6.6–7.6; Na+ 100–175 mmol L^−1^; K+ 2.0–6.0 mmol L^−1^	Electrolyte/hydration monitoring
[48]	Environmental/preventive sensing	Porcine skin ex vivo; human skin in vivo	Tattoo gun	Photochromic tattoo ink based on nanocapsules containing leuco dyes	Visual/naked-eye, optional image analysis	UV-dependent color change versus UVA/UVB intensity	UV exposure monitoring and skin-cancer prevention
[3]	Metabolite sensing	Porcine skin ex vivo; phantom tissue	Tattoo gun	Colorimetric biosensor formulations: methyl red, bromothymol blue, phenolphthalein, glucose oxidase, peroxidase, TMB, and albumin-responsive dye	Visual + smartphone-based	pH 5.0–9.0; glucose up to 50.0 mmol L^−1^; albumin up to 5.0 g L^−1^	Point-of-care metabolite monitoring
[4]	Electrical sensing/monitoring	Human on-skin testing; Sprague–Dawley rat in vivo	Tattoo gun for dermal part; aerosol printing for epidermal part	Conductive PPEL ink based on PEDOT:PSS, EG, and LiTFSI	Electrical	Conductivity 5165 S cm^−1^; impedance 10.6 kΩ cm^2^ at 100 Hz; stretchability 56.9%	ECG/EMG and in vivo electrophysiological recording
[5]	Treatment	Artificial skin, fish skin, rat skin; free-walking rat wound model	Tattoo gun	Commercial tattoo ink with Fe/MXene/Zn; glycerol:methanol-based Ink with Fe/MXene/Zn for bioresorbable formulations	Electrical/triboelectric	Triboelectric voltage generation; wound closure about 80% vs 60% control	Self-powered wound healing and therapeutic dermal tattoo bioelectronics

## 6 Advantages, challenges, and future perspectives of dermal tattoos in biomedicine

Dermal tattoos are emerging as a distinctive class of biomedical interface that occupies a unique position between conventional wearables and implantable systems. As shown by the current literature, they combine several unusual features within a single platform: access to the dermal microenvironment, long-term residence within the skin, compatibility with both sensing and bioelectronic functions, and, in some cases, aesthetic customization and bioresorbability. These properties make dermal tattoos conceptually attractive for next-generation biomedicine. At the same time, the field is still in an early stage, and important scientific, technological, regulatory, and translational challenges remain unresolved. Thus, the future of dermal tattoos in biomedicine will depend not only on demonstrating novel proof-of-concept systems, but also on establishing their safety, reproducibility, usability, and clinical relevance.

### 6.1 Opportunities and key advantages

One of the most important advantages of dermal tattoos is their stable integration with the skin (Table 3). Because the active material is embedded beneath the epidermis rather than adhered to the surface, dermal tattoos are less exposed to friction, sweat, and external contamination than epidermal wearables. This makes them especially attractive for applications requiring stable long-term sensing or durable tissue interfacing. Papers focused on colorimetric [44], fluorescent and optical sensing [47], repeatedly emphasize the value of the dermal location for maintaining access to interstitial fluid while protecting the sensing chemistry from environmental interference. This combination of physiological access and mechanical protection is one of the central reasons dermal tattoos differ fundamentally from surface-mounted systems.

**Table 3:** Key opportunities, current limitations, and future directions of dermal tattoos in biomedicine.

Aspect	Current opportunity/advantage	Current limitation/challenge	Future direction
**Skin integration**	Stable under-skin positioning and intimate tissue contact	Invasive placement compared with epidermal wearables	Improved minimally invasive delivery methods
**Signal stability**	Reduced exposure to sweat, friction, and surface contamination	Long-term functional drift still unclear	Long-duration validation in vivo
**Motion artifact**	Improved electrophysiological signal quality and higher SNR reported in dermal electrode demonstrations	Not yet broadly validated across device types	Standardized benchmarking versus epidermal systems especially on direct contact impedance
**Access to physiology**	Direct interaction with interstitial fluid and dermal tissue environment	Analyte transport and local variability may complicate interpretation	Better physiological correlation studies with blood and tissue markers
**Aesthetic compatibility**	Can be merged with artistic or discreet tattoo designs	User acceptance may vary across cultures and clinical contexts	Personalized and patient-specific biomedical tattoo designs
**Device lifetime**	Can support both persistent and transient designs	Difficult to precisely control retention or degradation in some systems	Programmable bioresorbable and time-tunable tattoo platforms
**Readout**	Compatible with visual, optical, smartphone-based, and electrical outputs	Calibration, lighting effects, and signal standardization remain problematic	Robust portable readers, AI-assisted analysis, and standardized software
**Functional diversity**	Applicable to sensing, monitoring, and emerging therapy	Therapeutic applications remain very limited	Expansion toward electroceuticals and closed-loop theranostic systems
**Materials**	Wide material toolbox: dyes, fluorescent probes, bacteria, conductive inks	Biocompatibility and long-term safety remain incompletely understood	Safer, validated, and clinically translatable material platforms
**Translation potential**	Intermediate platform between wearables and implants	Regulatory classification and manufacturing standardization remain unclear	Dedicated regulatory pathways and scalable manufacturing protocols

A related advantage is the potential for reduced motion artifact and improved interface quality. In the case of electrophysiological monitoring [4], under-skin tattoo electrodes were shown to reduce the unfavorable effects of the stratum corneum and improve signal acquisition relative to conventional on-skin electrodes. More broadly, deeper placement within the skin may reduce interface instability and help maintain more consistent contact with tissue during motion. This is particularly valuable in applications where signal quality is easily degraded by mechanical displacement or variable skin contact.

**Fig. 9:**
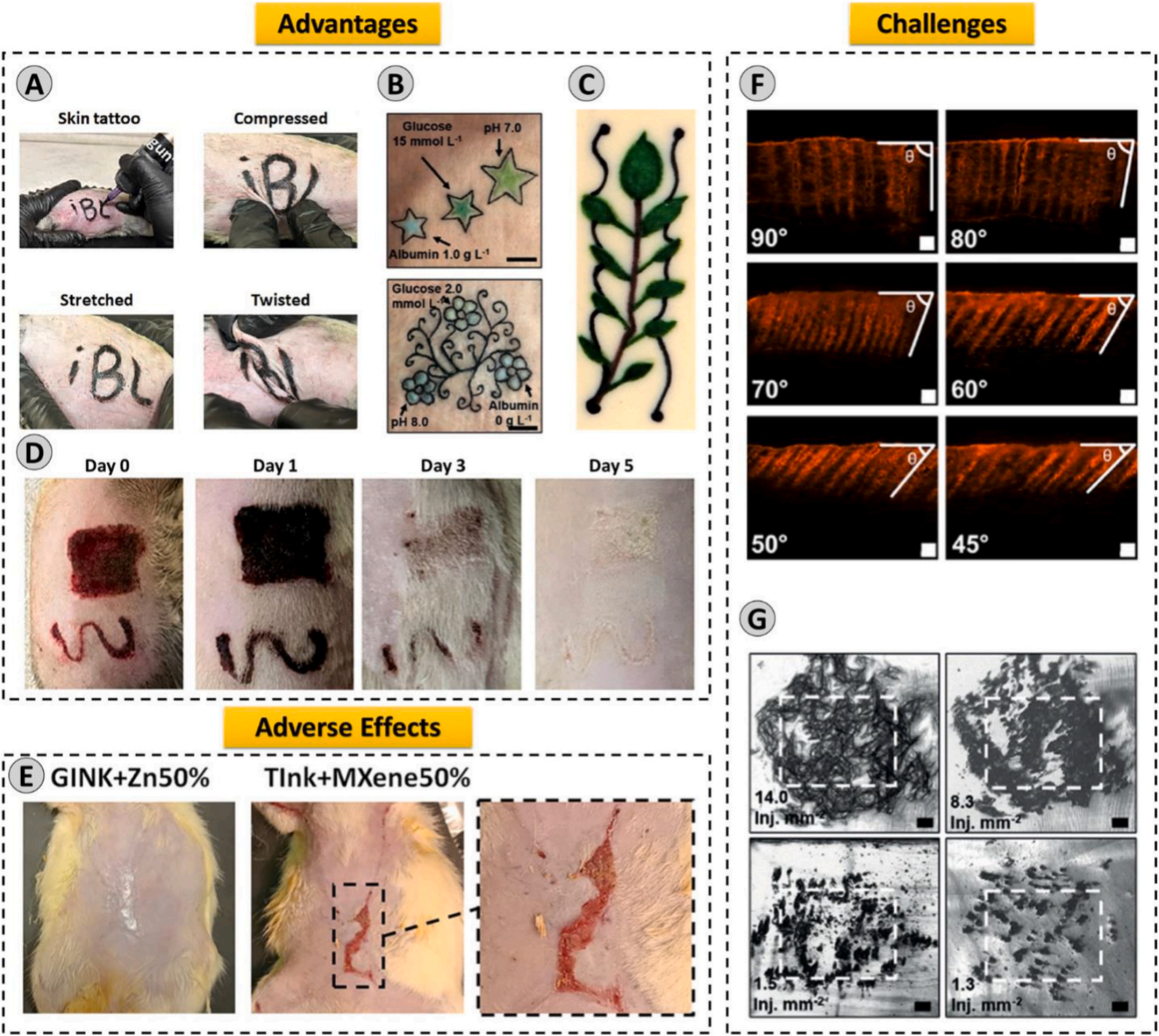
Representative advantages, challenges, and adverse effects of dermal tattoo platforms. (A) Mechanical adaptability of dermal tattoos, showing preservation of the tattoo pattern under compression, stretching, and twisting [5]. (B–C) Examples of aesthetic compatibility, illustrating functional dermal tattoos integrated into decorative or personalized tattoo patterns [3,5]. (D) Example of transient/bioresorbable tattoo behavior, showing progressive fading of the tattoo over time [5]. (E) Representative adverse effects observed with certain high-concentration formulations, including visible skin irritation and damage [5]. (F) Cross-sectional images showing how tattoo-gun insertion angle influences dermal deposition pattern and contributes to fabrication variability [47].(G) Examples of how injection density affects tattoo distribution and pattern uniformity, highlighting reproducibility challenges in dermal tattoo fabrication [3].

Another important advantage of dermal tattoos is their mechanical adaptability to the body. Because the tattooed patterns are embedded within soft tissue rather than mounted as rigid external components, they can conform to curvilinear and irregular body surfaces while remaining mechanically integrated with the skin. As demonstrated in recent work [5], dermal tattoo patterns can remain functional while being compressed, stretched, or twisted (Fig. 9-A), highlighting their ability to tolerate routine body deformation without losing their structural continuity or biomedical function. This mechanical compliance is particularly valuable for applications on mobile anatomical regions, where conventional surface-mounted devices may detach, slip, or suffer from signal instability.

Dermal tattoos also offer a notable degree of aesthetic compatibility (Fig. 9-B and -C) and user-centered personalization [3,5]. Unlike many conventional biomedical devices, which remain visibly medical and may feel cumbersome or stigmatizing, dermal tattoos can potentially be merged with artistic designs or made visually subtle, as shown by both decorative-photoresponsive concepts and the treatment-oriented studies. This characteristic may be especially important in long-term or visible applications, where patient comfort is shaped not only by physical sensation but also by appearance, identity, and social acceptability. The possibility of combining function with body art may therefore become one of the defining advantages of dermal tattoo medicine.

Another major opportunity is the flexibility of dermal tattoos to support either long-term or transient use, depending on the functional material. Some platforms are intentionally designed for prolonged retention and monitoring, such as photochromic UV tattoos or stable dermal sensing chemistries, while others can be engineered to degrade naturally after use, as demonstrated by the bioresorbable Zn-based therapeutic tattoo system (Fig. 9-D) [5]. This ability to tune the device lifetime to the clinical need is highly attractive. It suggests that dermal tattoos could serve both as long-term monitoring interfaces and as short-lived treatment systems that do not require removal procedures.

Finally, dermal tattoos provide a compelling form of biointegration with relatively low user burden. They are more deeply integrated than conventional wearables, yet can remain more conformal and less intrusive than many implanted systems. This positions them as a promising intermediate platform for future point-of-care medicine, especially in settings where repeated sampling, continuous monitoring, or localized treatment is desired. Taken together, these features suggest that dermal tattoos are not simply a variation of epidermal devices, but rather a distinct biomedical platform with its own set of advantages.

### 6.2 Current limitations and challenges

Despite their promise, dermal tattoos still face substantial limitations (Table 3). The first and perhaps most important challenge is biocompatibility and possible adverse effects of the inks. Because these systems rely on introducing functional materials directly into living tissue, their safety requirements are closer to those of implantable or tissue-contacting biomaterials than to many surface-mounted wearables. Therefore, the intended residence time of the tattoo material in the dermis is a key consideration [71,72]. Short-term or transient systems may use degradable materials such as polylactic acid (PLA) [73,74], poly(lactic-co-glycolic acid) (PLGA) [[75], [76], [77]], or hydrogel-based matrices that gradually degrade and are cleared from the body [78,79], whereas long-term systems require highly stable and biocompatible materials, similar in principle to established implantable biomaterials such as titanium, platinum, or medical-grade silicone [[80], [81], [82], [83], [84]]. In dermal tattoo technologies, two broad material strategies can be considered. The first involves the modification of commercially available tattoo inks that are already used in cosmetic tattooing; however, once functional additives such as nanoparticles, sensing dyes, conductive materials, or photoresponsive components are incorporated, their toxicity, immune response, migration, degradation, and long-term stability must be carefully reassessed [71,[85], [86], [87], [88], [89], [90]]. The second strategy involves newly fabricated functional inks or biomaterial systems specifically designed for biomedical tattoo applications, which require comprehensive evaluation of cytotoxicity, inflammatory response, degradation products, clearance, and chronic biocompatibility before translation. This issue becomes especially important when the tattoo ink is based on advanced materials including nanoparticles, engineered bacteria, conductive polymer blends, photochromic nanocapsules, or persistent sensing chemistries [45,55].

There are also reports raising concerns about adverse effects associated with dermal tattoo materials, particularly when high concentrations of a core functional component are used, as illustrated in Fig. 9-E [5]. Although several studies have reported promising biocompatibility or successful implantation in skin models and animal systems, broad long-term evidence is still lacking regarding chronic inflammatory responses, degradation behavior, material migration, and long-term tissue tolerance across different material classes. Material concentration is especially important in this context. For example, the work published by Shakibi et al. [5] using MXene-based conductive materials incorporated into tattoo inks, low concentrations did not produce detectable cytotoxicity, whereas higher concentrations caused measurable tissue damage. This finding highlights the need for dose-dependent toxicity evaluation when integrating functional nanomaterials, conductive additives, or other active components into dermal tattoo systems.

Tatoo inks composition is often complex and insufficiently regulated, with some inks containing metallic impurities, azo pigments, polycyclic aromatic hydrocarbons, phenols, phthalates, or other potentially toxic compounds [[91], [92], [93]]. Recent analytical studies further emphasize the need for regulatory oversight and chemical characterization, as some commercial tattoo inks have been reported to contain toxic metals, carcinogenic aromatic amines, or preservatives exceeding regulatory limits, highlighting the importance of validated analytical methods, accurate labelling, and strict quality control before adapting such materials for biomedical applications [94,95]. Some pigments may also degrade under ultraviolet or laser exposure, producing byproducts with possible cytotoxic, genotoxic, or carcinogenic effects [91,96,97]. In the skin, tattoo pigments are not completely inert; they can trigger chronic inflammatory or foreign-body responses and may be taken up by macrophages and transported to regional lymph nodes [98,99]. Clinically, tattoo inks have been associated with allergic reactions, granulomatous inflammation, infections, pseudoepitheliomatous hyperplasia, and rare reports of tumors arising within tattoos [[100], [101], [102], [103]]. However, a direct causal link between tattooing and cancer has not been clearly established, and current evidence remains limited. These findings highlight the need for better regulation, compositional transparency, and long-term in vivo studies to evaluate the safety of tattoo inks, particularly when they are modified with functional materials for biomedical applications [103,104].

Tattooing can cause bacterial infections due to contaminated inks or poor hygiene during the procedure. Reported complications include local skin infections (e.g., abscesses, cellulitis) and severe systemic infections such as endocarditis or septic shock. Studies also show that some tattoo inks may contain bacteria, making contamination a potential source of infection [86,105,106].

Tattooing can trigger immune and inflammatory responses because the skin is injured by repeated needle punctures and exposed to foreign ink materials [98]. A common short-term reaction is transient edema, which may occur in up to one-third of tattooed individuals and appears as swelling or hardening of the tattooed area, sometimes with itching, pigmentation changes, or minor bleeding [107]. This usually resolves within 2–3 weeks and is mainly caused by mechanical trauma, irritation, and acute inflammation against the ink or diluent [108,109]. Tattoo inks may also cause allergic or immune-mediated reactions, such as allergic contact dermatitis, presenting as redness, vesicles, scaling, thickened skin, or ulcer-like lesions. These reactions can be enhanced by UV exposure, which may chemically alter tattoo pigments through haptenization, making them recognizable to the immune system as foreign substances [90,110]. Red inks are most commonly associated with hypersensitivity reactions, historically linked to mercury-containing pigments. However, identifying the exact allergen is often difficult because tattoo ink compositions are not always fully disclosed and patch testing is frequently negative [111,112].

Tattoo-related chronic toxicity may occur through persistent immune stimulation by retained pigments, leading to delayed complications such as granulomatous inflammation, lichenoid reactions, pseudolymphoma, fibrosis/morphea-like changes, and flare-ups of preexisting skin diseases such as psoriasis, vitiligo, lupus, or lichen planus. These reactions can appear months to years after tattooing and may reflect long-term foreign-body responses or hypersensitivity to ink components [49,101,113,114].

In addition to biological safety, regulatory and clinical translation of dermal tattoo biomedicine will require clearer classification and validation pathways. These systems may not fit neatly into a single conventional category, because depending on their design they may function as diagnostic devices, implantable materials, drug–device combinations, tattoo products, bioelectronic interfaces, or even living engineered systems. Therefore, regulatory evaluation should be based on the intended function, duration of dermal residence, material composition, and level of interaction with tissue. For biomedical dermal tattoos, the ISO 10993 series [115] is particularly relevant because functional inks, nanoparticles, hydrogels, conductive polymers, fluorescent probes, or bioresorbable components are placed in direct contact with dermal tissue. Biological evaluation may therefore need to include cytotoxicity, irritation, sensitization, local tissue response after implantation, systemic toxicity, chemical characterization, extractables/leachables, degradation products, and long-term retention or clearance.

Beyond biocompatibility, translation will also require reproducible manufacturing under appropriate quality systems, including control of ink composition, particle size, rheology, sterility, tattooing depth, dose of delivered material, and batch-to-batch consistency. For active electrical or optoelectronic dermal tattoos, additional validation will be needed for electrical safety, signal reliability, software or reader performance, packaging, and stable connection to external electronics [116].

Furthermore, clinical translation should be application-driven: dermal tattoos must demonstrate not only feasibility, but also clear practical benefit over existing epidermal wearables, conventional biosensors, or implantable devices. Future validation pathways should therefore include standardized preclinical testing, long-term in vivo safety studies, comparison with established diagnostic or therapeutic standards, and eventually human studies evaluating accuracy, durability, usability, patient acceptance, and healthcare workflow integration.

Another major challenge is standardization and reproducibility of the tattooing. Current dermal tattoo studies mainly rely on two fabrication methods, microneedles and conventional tattoo guns, and these approaches differ substantially in their degree of control. In both cases, factors such as penetration depth, local material distribution, and pattern uniformity can directly influence sensing performance or electrical behavior. However, this variability is especially pronounced in tattoo-gun-based methods, where key parameters such as insertion angle (Fig. 9-F), penetration depth, tattooing density (Fig. 9-G), and even local ink distribution depend strongly on the operator and can therefore affect the final dermal architecture and functional output [3,47]. Additional factors such as ink rheology and particle sedimentation may further amplify this variability. By contrast, microneedle-based approaches generally provide a more controlled and reproducible route, with better-defined insertion geometry and spatial patterning. Because the field is still young and methodologically diverse, there is little standardization in how dermal tattoo systems are fabricated, characterized, and compared. This remains a major barrier to reproducibility across laboratories and to future regulatory approval.

Readout reliability is another challenge. Many current dermal tattoo systems, especially colorimetric and optical platforms, remain at least partly dependent on lighting conditions, camera settings, imaging angle, or user handling. While smartphone integration improves accessibility, it also introduces variability related to hardware differences, calibration, and image processing. Even fluorescence-based systems that use dedicated readers may face signal drift, optical instability, or sensitivity to environmental conditions. Electrical systems, in turn, face their own issues, including noise management, signal stability, and long-term maintenance of conductive interfaces. Thus, readout is not merely a convenience issue but a core translational challenge that must be addressed if dermal tattoo systems are to become reliable clinical tools.

Despite the potential of dermal tattoo biosensors, several biological and physical challenges must be addressed to translate these systems into clinical practice. From a biological perspective, the complex environment of the dermis characterized by dynamic interstitial fluid (ISF) composition, potential physiological lag between blood and ISF, and the host's immune responses, such as inflammation and long-term tissue remodeling requires further systematic investigation. Furthermore, the impact of biofouling on long-term sensor stability and the repeatability of enzymatic or chemical sensing reactions remains largely unexplored. From an optical and physical standpoint, developing reliable readout methods is hindered by real-world variables, including the influence of skin tone and melanin on signal attenuation, hemoglobin absorption, tissue scattering, and varying tattoo depths. Additionally, ambient lighting conditions pose significant challenges for non-laboratory quantification, underscoring the need for the integration of internal references or ratiometric calibration strategies to ensure measurement accuracy. Addressing these factors specifically through comparative studies with established fields like microneedle-based sensing and implantable electronics is essential to advance the reliability and clinical utility of dermal tattoo-based biosensing.

While dermal tattoo–based electrophysiological platforms show potential, their development faces significant technical hurdles. Ensuring long-term electrical stability in vivo remains challenging due to potential material degradation, biofouling, and inflammatory responses at the electrode–tissue interface. Furthermore, robust interconnection and packaging strategies are required to manage signal routing from the dermis to external electronics while minimizing signal noise and drift. Given the nascent state of this field, there is currently a lack of comprehensive long-term in vivo data and systematic benchmarking against state-of-the-art epidermal or implantable electrodes. Addressing these critical research gaps regarding system integration, robustness, and signal fidelity is essential for the future translational viability of these technologies.

User acceptance and patient perception also represent important but often overlooked challenges for dermal tattoo-based biomedical technologies. Beyond technical performance, the adoption of such systems may be influenced by cultural and religious attitudes toward tattooing, which vary widely across different societies and may limit acceptance in certain populations. Additional concerns include fear of needles and pain during application, the perceived permanence of tattoos, and the visibility of health-related signals on the skin, which may raise privacy issues or potential social and professional stigma. At present, however, the literature addressing these sociocultural and patient-centered aspects of biomedical tattoo technologies remains unstudied. Therefore, future studies should investigate patient perception, usability, and cultural acceptance to better understand how these factors may influence the real-world implementation of dermal tattoo-based biomedical platforms.

Finally, an additional limitation is the small size of the field itself. Although dermal tattoos are conceptually rich and multidisciplinary, the published biomedical literature remains very limited. Most studies are still proof-of-concept demonstrations, and the majority focus on sensing [3,44,46] rather than treatment [5]. Only a small number move into bioelectronics [4], and even fewer into therapy [5]. As a result, the field still lacks the breadth of independent validation and comparative benchmarking needed to establish mature design rules or clinical priorities. This scarcity also means that many promising directions, such as closed-loop systems or broader therapeutic applications, remain largely speculative for now.

### 6.3 Future perspectives

Looking ahead, dermal tattoos have the potential to evolve into a new class of personalized and multifunctional biomedical interfaces [55]. One promising direction is the development of theranostic tattoos, in which sensing and treatment are combined within the same platform. At present, sensing and therapy remain largely separate branches of the literature, but the emergence of self-powered therapeutic tattoos suggests that future systems could move toward closed-loop operation, where a tattoo first detects a physiological state and then triggers or modulates a local therapeutic response. This would represent a major conceptual advance in skin-integrated medicine.

Another important opportunity lies in personalization and digital integration. Several existing dermal tattoo platforms already rely on smartphone imaging, app-based analysis, or wearable optical readers. These features could be extended further through AI-assisted image interpretation, individualized calibration, cloud-based monitoring, and remote healthcare connectivity. Because dermal tattoos can remain in place for prolonged periods, they are particularly well suited to longitudinal and personalized monitoring strategies. In parallel, the possibility of aligning tattoo geometry with patient preference or artistic design may make future systems more acceptable and adaptable than conventional medical hardware.

The treatment side of the field also holds major promise. At present, wound healing is the only clearly demonstrated therapeutic direction in the current paper set, but the broader concept of dermal tattoo electroceuticals could expand to areas such as regenerative medicine, localized stimulation, or short-term bioelectronic therapy. In this context, bioresorbable materials may become especially important, allowing therapeutic tattoos to disappear naturally after the intervention is complete. Such transient platforms would align well with the needs of post-surgical care, temporary stimulation, and other limited-duration interventions.

Another promising yet underexplored direction is the incorporation of electrochemical sensing into dermal tattoos. By integrating conductive inks or microelectrode-based interfaces capable of interacting with interstitial fluid, these platforms could enable real-time, quantitative monitoring of metabolites and electrolytes, offering an alternative to current optical readout approaches. Although such systems remain largely unreported, their combination with flexible and skin-compatible electronics represents a compelling future pathway.

Because the dermal tattoo field is still small, future studies should selectively borrow evaluation criteria from more mature adjacent fields. Microneedle-based ISF sensors and continuous glucose monitoring systems provide useful benchmarks for analyte transport, local ISF–blood correlation, physiological lag, and calibration requirements [117,118]. Implantable biosensors highlight the importance of biofouling, protein adsorption, foreign-body response, fibrotic encapsulation, long-term drift, and tissue response, all of which may also affect functional materials retained in the dermis [119,120]. Epidermal electronics provide relevant benchmarks for conformability, adhesion, motion artifacts, interfacial noise, and signal quality during daily activity [121,122]. Therefore, dermal tattoo platforms should not be evaluated in isolation; their future validation should incorporate transport analysis from microneedle and CGM research, drift and biofouling assessment from implantable biosensors, and motion-artifact benchmarking from epidermal electronics.

Overall, the future of dermal tattoos in biomedicine will likely depend on moving from isolated proof-of-concept studies toward standardized, clinically driven, and multifunctional systems. If current challenges in safety, fabrication, readout, and regulation can be addressed, dermal tattoos may ultimately become a uniquely powerful biomedical platform (Table 3) combining the intimacy of skin integration, the versatility of advanced materials, and the possibility of both personalized sensing and localized therapy.

## 7 Conclusion

Dermal tattoos are emerging as a distinctive biomedical platform that bridges the gap between conventional devices, wearable systems, and deeper skin-integrated interfaces. Although the field is still in its early stages and currently dominated by sensing applications, the available studies collectively demonstrate that dermal tattoos can support a remarkably broad range of functions, including colorimetric, fluorescent, living, and electrophysiological sensing, as well as early therapeutic applications such as self-powered wound healing. Their unique combination of stable dermal integration, access to interstitial fluid, reduced exposure to surface contamination, and compatibility with both long-term and transient designs gives them clear promise for future personalized medicine. At the same time, important challenges remain, particularly in biocompatibility, fabrication standardization, reproducibility, readout reliability, and clinical translation. As materials, tattooing strategies, and bioelectronic technologies continue to advance, dermal tattoos may evolve from proof-of-concept systems into multifunctional and theranostic biomedical interfaces that combine sensing, monitoring, and treatment within a single, minimally intrusive platform.

## Funding

The authors declare that no funds, grants, or other support were received for this work.

## CRediT authorship contribution statement

**Reyhaneh Shakibi:** Data curation, Formal analysis, Investigation, Methodology, Project administration, Software, Validation, Visualization, Writing – original draft, Writing – review & editing. **Ali Golkar:** Formal analysis, Investigation, Writing – review & editing. **Hamid Reza Bagheri:** Data curation, Formal analysis, Investigation, Methodology. **Mohammad Ali Khayamian:** Conceptualization, Data curation, Formal analysis, Funding acquisition, Investigation, Methodology, Project administration, Resources, Software, Supervision, Validation, Visualization, Writing – original draft, Writing – review & editing.

## Declaration of competing interest

The authors declare that they have no known competing financial interests or personal relationships that could have appeared to influence the work reported in this paper.

## Data Availability

Data will be made available on request.
